# TACOA – Taxonomic classification of environmental genomic fragments using a kernelized nearest neighbor approach

**DOI:** 10.1186/1471-2105-10-56

**Published:** 2009-02-11

**Authors:** Naryttza N Diaz, Lutz Krause, Alexander Goesmann, Karsten Niehaus, Tim W Nattkemper

**Affiliations:** 1 Center for Biotechnology (CeBiTec), Bielefeld University, Bielefeld, Germany; 2 Biodata Mining & Applied Neuroinformatics Group, Faculty of Technology, Bielefeld University, Bielefeld, Germany; 3 Proteome and Metabolome Research, Faculty of Biology, Bielefeld University, Bielefeld, Germany; 4 Computational Genomics, Center for Biotechnology (CeBiTec), Bielefeld University, Bielefeld, Germany; 5 Nestlé Research Center, BioAnalytical Science Department, Lausanne, Switzerland

## Abstract

**Background:**

Metagenomics, or the sequencing and analysis of collective genomes (metagenomes) of microorganisms isolated from an environment, promises direct access to the "unculturable majority". This emerging field offers the potential to lay solid basis on our understanding of the entire living world. However, the taxonomic classification is an essential task in the analysis of metagenomics data sets that it is still far from being solved. We present a novel strategy to predict the taxonomic origin of environmental genomic fragments. The proposed classifier combines the idea of the *k*-nearest neighbor with strategies from kernel-based learning.

**Results:**

Our novel strategy was extensively evaluated using the leave-one-out cross validation strategy on fragments of variable length (800 bp – 50 Kbp) from 373 completely sequenced genomes. TACOA is able to classify genomic fragments of length 800 bp and 1 Kbp with high accuracy until rank class. For longer fragments ≥ 3 Kbp accurate predictions are made at even deeper taxonomic ranks (order and genus). Remarkably, TACOA also produces reliable results when the taxonomic origin of a fragment is not represented in the reference set, thus classifying such fragments to its known broader taxonomic class or simply as "unknown". We compared the classification accuracy of TACOA with the latest intrinsic classifier PhyloPythia using 63 recently published complete genomes. For fragments of length 800 bp and 1 Kbp the overall accuracy of TACOA is higher than that obtained by PhyloPythia at all taxonomic ranks. For all fragment lengths, both methods achieved comparable high specificity results up to rank class and low false negative rates are also obtained.

**Conclusion:**

An accurate multi-class taxonomic classifier was developed for environmental genomic fragments. TACOA can predict with high reliability the taxonomic origin of genomic fragments as short as 800 bp. The proposed method is transparent, fast, accurate and the reference set can be easily updated as newly sequenced genomes become available. Moreover, the method demonstrated to be competitive when compared to the most current classifier PhyloPythia and has the advantage that it can be locally installed and the reference set can be kept up-to-date.

## Background

Metagenomics, or the direct sequencing of collective genomes is paving the road to a better understanding of our ecosystems and the impact of microbes on human health. Researchers are now changing the genome-centric approach, which focussed on isolation, cultivation and sequencing of single species at a time by sequencing complete DNA samples from an environment, thus bypassing the isolation and cultivation step. At present, most metagenomes are sequenced using the whole genome shotgun approach [1]. When used in combination with the Sanger technique [2,3], a collection of short sequence *reads *with average length of 800 bp is generated [4]. Recovery of DNA fragments of several thousand base pairs is also possible using bacterial artificial chromosomes (BACs) [5]. Longer DNA fragments can be also obtained when short overlapping reads are assembled into larger DNA stretches referred to as *contigs*.

An essential task addressed in the metagenomic data analysis workflow is to predict the source organism or *taxonomic origin *of each read or assembled contig. This process is called taxonomic classification or *binning*. Predicting the taxonomic origin of reads or contigs can aid in linking gene functions to members of the community or to reconstruct the microbial composition of the studied sample. The knowledge of the taxonomic composition of a sample can be used to derive valuable ecological parameters at the community level (e.g. richness and evenness) [6,7] or at the population level (e.g. effective genome size) [8].

Two types of methods are used for the taxonomic classification of environmental fragments: Composition-based and similarity-based-methods. Similarity-based-methods depend on a sequence-comparison with a reference set of genomic sequences. Similarity-based methods directly align metagenomic sequences to a reference set, e.g. using BLAST [9]. Composition-based methods rely on characteristics that can be extracted directly from the nucleotide sequences (e.g. oligonucleotide frequencies, GC-content, etc.). Recently, methods employing sequence-composition-based features are gaining popularity [10-13]. In particular, oligonucleotide frequencies have frequently been used because they carry a phylogenetic signal [14,15]. Karlin *et al*. [14] showed that significant deviations in terms of di-nucleotide or tetra-nucleotide frequencies were less significant within a genome than between genomes of different species.

From a machine learning point of view composition- and similarity-based methods can be further divided into supervised and unsupervised apporaches. In the context of this work, supervised methods require a reference set of genomic sequences with known taxonomic origin. Supervised composition-based methods use the reference set to learn sequence characteristics of each taxonomic class during a training phase. Subsequently, the trained classifier is used to identify the taxonomic class of fragments of unknown origin. For example methods such as a Bayesian classifier [16] and PhyloPythia [12] fall into the supervised composition-based category. Although MEGAN [17] and CARMA [6] do not have a training phase, these similarity based classifiers are supervised since they rely on the alignment of the genomic fragments to reference sequences with known taxonomic origin.

The recently published CARMA software [6] has been developed to taxonomically classify short reads (80 bp – 400 bp) derived by the Pyrosequencing technique (454 – Life Sciences) [18]. CARMA showed to be very accurate on taxonomically classifying reads that carry a complete or partial protein family contained in the Pfam database [19]. CARMA has the advantage of giving very accurate predictions but it is computationally expensive. MEGAN [17] performs well in classifying genomic fragments if closely related reference genomes are available, which may not be always the case for organisms contained in an environmental sample. In general, sequence similarity based classifiers, such as CARMA and MEGAN, have the disadvantage of being able to predict the taxonomic class for only those fragments carrying a partial gene or a protein domain. Compared to MEGAN and CARMA, our proposed strategy has the advantage of being easy to maintain and the complete strategy can be run on a desktop computer in a reasonable time frame without preprocessing steps. PhyloPythia, a supervised composition-based method, uses over-represented oligonucleotide patterns as features to train a hierarchical collection of Support Vector Machines (SVMs), which is subsequently used to predict the taxonomic origin of genomic fragments as short as 1 Kbp [12]. Support Vector Machines demonstrated to achieve a high classification accuracy for fragments of length ≥ 3 Kbp and moderate accuracy for 1 Kbp long fragments. However, the complete classifier needs to be retrained (a computationally expensive procedure) when newly sequenced genomes are added to the training set.

Unsupervised learning approaches do not depend on reference sequences for classification, instead characteristics are directly learned from the same data set that is being analyzed. In the context of metagenomics, unsupervised learning methods are used to group genomic sequences such that all sequences originating from the same taxon are grouped into one cluster. Notably, this grouping can be done on different taxonomic ranks, ranging from superkingdom to species. Unsupervised methods are for example employed as a pre-processing step for assembly or to study the community composition of samples. Additionally, marker sequences of known taxonomic origin can be used to infer the taxonomic origin of each generated cluster. However, in this case the marker sequences are not involved in the classification process *per se *[13].

Several unsupervised methods have been developed for the analysis of metagenomic data, the pioneering TETRA [20,21] used tetranucleotide-derived z-score correlations to taxonomically classify genomic fragments from metagenome libraries of low diversity. Abe *et al*. [10,11], in a following work, showed the feasibility to classify environmental genomic fragments with minimal length of 5 Kbp using a self-organizing map (SOM). More recently, Chan *et al*. developed a seeded growing self-organizing map (S-GSOM) [13] to cluster metagenomic sequences.

Currently, completely sequenced genomes, which could be used as a reference for the taxonomic classification of metagenomic sequences, become available at an exponential rate. Therefore, the taxonomic classification of metagenomic data will greatly benefit from supervised methods that can be instantaneously updated when new genomes become available. Herein, we present a TAxonomic COmposition Analysis method (TACOA) able to predict the taxonomic origin of environmental genomic fragments of variable length in a supervised manner. TACOA can be easily installed and run on a desktop computer offering more independence in the analysis of metagenomic data sets. Furthermore, the reference set used by the proposed classifier can easily be updated with newly sequenced genomes.

TACOA applies the intuitive idea of the *k*-nearest neighbor (*k*-NN) approach [22] and combines it with a smoother kernel function [23,24]. Compared to other less intuitive and more complex approaches, *k*-NN based methods have proven to yield competitive results in a large number of classification problems [25-28]. In particluar, if the classification problem has a multi-class nature. The kernelized *k*-NN approach used in TACOA allows to realize an accurate multi-class classifier. In general, *k*-NN is intuitive, does not make any assumptions about the distribution of the input data and the reference set can be easily updated. For a wide range of practical applications it approximates the optimal classifier if the reference set is large enough. A further advantage is that the classification results can be easily interpreted. However, the traditional *k*-NN algorithm runs into problems when dealing with high dimensional input data (called curse of dimensionality) [23]. In our extension of *k*-NN, the introduction of a Gaussian kernel helps to alleviate this problem. [23]. By using a smoother kernel function the complete reference set is considered during the classification procedure instead of a strict neighborhood. We present our kernelized *k*-NN approach as an alternative to solve the problem of taxonomically classifying environmental genomic fragments.

## Results

The idea behind our approach is to exploit the benefits of the case-based-reasoning *k*-NN algorithm, which classifies vectors (i.e. Genomic Feature Vectors, GFVs) on the basis of the class labels observed for vectors in its neighborhood while keeping the advantage to approximate to the optimal classifier if the training set is large enough. In particular, we used a smoother kernel function with Gaussian density to profit from its implicit weighting scheme, thus allowing more flexibility on setting the neighborhood width and in handling high-dimensional input data. The weights given by a smoother kernel function decrease as the Euclidean distance between the classified GFV and the reference vector increases. The rate at which the weights decreases is controlled by the neighborhood width *λ *[23].

### Algorithm

In this study, a genomic fragment is defined as a DNA sequence of a given length (note, that a completely sequenced genome can be regarded as a genomic fragment). The total number of oligonucleotides of length *l*, from the alphabet ∑ = {*a*, *t*, *c*, *g*} is given by 4^*l*^. Each genomic fragment is represented as a vector (i.e. GFV) using the Vector Space Model [29]. For each of the possible four oligonucleotides in a sequence, the vector stores the ratio between the observed frequency of that oligonucleotide to the expected frequency given the GC-content of that genomic fragment.

In order to predict the taxonomic origin of a query GFV, TACOA compares that query GFV to the reference GFVs. In our method, the reference GFVs are computed from all 373 completely sequenced reference genomes. In the following, the set of all computed reference GFVs is named as reference set (**ref**_*set*_). In this study a reference set consisting of 373 genomes was used, i.e. *T *= 373 in this case.

More formally, let **ref**_*set *_= {**x**_*j *_} with 1 ≤ *j *≤ *T *be the set of reference GFVs, where each **x**_*j *_represents a GFV computed from a completely sequenced reference genome. Let **x **be a query GFV representing a genomic fragment to classify. The multi-class classification problem addressed herein, resides in deciding to which of all different taxonomic classes, at rank *r*, **x **belongs to.

For each taxonomic rank *r *out of superkingdom, phylum, class, order and genus and for each taxonomic class *i *at that rank, the algorithm computes a discriminant function *δ*_*i*_(**x**), and then classifies **x **into that class with the highest value for its discriminant function.

More precisely, for a given taxonomic rank *r*, let *i *be that class with the highest discriminant function *δ*_*i*_(**x**). Then, **x **is classified into class *i *if *δ*_*i*_(**x**) is at least half as large as the value of the second highest discriminant function on rank *r*, otherwise **x **is classified as "unclassified". This optimal cut-off value for the discrimination function at each taxonomic rank *r *was identified in a grid search. The discriminant function for a taxonomic class *i *is computed by:

(1) $$\delta_{i}(x)=\sum_{x_{j}\in ref_{i}}K_{\lambda }(x,x_{j})$$

where **ref**_*i *_= {**x**_*j*_|**x**_*j *_∈ **ref**_*set *_and **x**_*j *_stems from class *i*} is the set of all reference GFVs from class *i*. The smoother kernel *K*_*λ*_(**x**, **x**_*j*_) is based on the Gaussian density function that exponentially decreases with Euclidian distance from **x**:

(2) $$K_{\lambda }(x,x_{j})=e^{\left(-\frac{d_{w}{\left(x,x_{j}\right)}^{2}}{2\lambda }\right)}$$

where *d*_*w*_(**x**, **x**_*j*_) is a weighted distance function as defined later in Equation (4) and *λ *controls the neighborhood width around **x **in the kernel function. Small values of *λ *result in decision boundaries with higher variance that well-fit the reference set while large values achieve smooth and stable decision boundaries that avoid overfitting and are more robust [23].

In order to estimate how much a query GFV **x **differs from a reference GFV the distance between the two vectors is determined. By normalizing each vector to unit length differences in genomic vector lengths are corrected. The distance *d *between a query GFV **x **and each reference GFV **x**_*j *_is computed using the dot-product between the normalized query GFV $\hat{x}$ and the normalized reference GFV ${\hat{x}}_{j}$:

(3) $$d(x,x_{j})=1-<\hat{x},{\hat{x}}_{j}>$$

The distance *d *was weighted in order to account for the imbalanced reference set used in this study, where majority classes and minority classes are present, e.g. the bacteria group is over-represented compared to the archaea in a proportion of 10:1.

The weighted distance function is denoted as *d*_*w *_and the weights are assigned using the following weighting scheme. Let **x**_*j *_originate from class *i *and let *n*_*i *_be the number of genomes in class *i*. Furthermore, let *T *be the number of genomes constituting the reference set. The weighted distance function *d*_*w *_is given by:

(4) $$d_{w}(x,x_{j})=\frac{T}{n_{i}}d(x,x_{j})$$

This weighting scheme assigns small weights to the GFVs belonging to the majority classes and a relative larger weight for GFVs contained in the minority classes.

### Testing

As a proof of concept the method was evaluated on a data set containing fragments from 373 completely sequenced genomes representing a vast majority of members from the archaeal and bacterial group. All completely sequenced genomes available up to March 2008 were downloaded from the SEED database [30]. The selected genomes represent 2 Superkingdoms, 11 Phyla, 21 Classes, 45 Orders and 61 Genera. The taxonomic information for this data set was collected from the taxonomy database located at the US National Center for Biotechnology Information (NCBI) [31]. Some of the genomes downloaded from SEED were unfinished and present as several contigs. In this case, all contigs of each genome were arbitrarily joined together.

### Evaluation strategy

**Figure 1:**
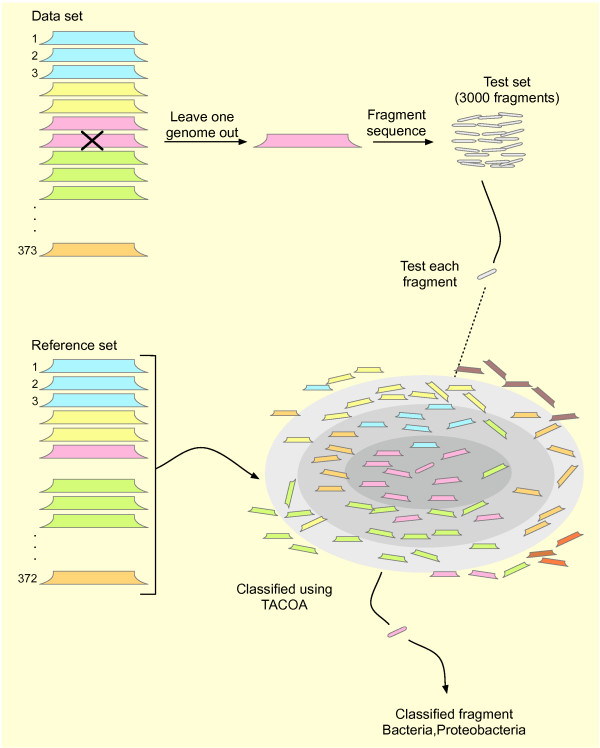
**A sketch of the leave-one-out cross validation strategy adopted in this study**. A genome is selected from the data set comprising 373 genomes and fragmented subsequently. The collection of genomic fragments is regarded as the test set from which each fragment is drawn and subsequently classified. Classification of each test fragment is carried out using the remaining 372 organisms as a reference.

The classification accuracy of the presented method was assessed using the leave-one-out cross-validation strategy. In the leave-one-out cross validation, one genome is used to generate fragments of a fixed length and thereafter the taxonomic origin of each fragment was predicted using the remaining 372 genomes and used as the reference set (Figure 1). This simulates the case when the taxonomic origin of DNA fragments is predicted that stem from genomes that are not yet represented in the public genome databases. In a second experiment we also evaluated the classification accuracy of the method with the test set included in the reference set, i.e. in this case the fragments of each genome were taxonomically classified using all 373 genomes as a reference. This experiment simulated the case when fragments need to be classified but they stem from genomes that are already represented in the reference set.

### Parameter optimization

We extensively investigated the oligonucleotide length parameter choosing different values of *l *(2 ≤ *l *≤ 6) and detected the length which resulted in the maximal classification accuracy. For short fragment lengths only small *l *values were considered to guarantee that all possible oligonucleotides have a sufficient occurrence, i.e. 4^*l *^< |*s*| in a genomic fragment *s *(see Methods). The optimal oligonucleotide length *l *was identified for each genomic fragment length at each taxonomic rank.

Oligonucleotides of length 4 were sufficient to achieve high classification rates for genomic fragments of length 800 bp, 1 Kbp, and 3 Kbp. For genomic fragments of length 10 Kbp, 15 Kbp, and 50 Kbp, oligonucleotides of length 5 were best suited for classification. A general trend for all genomic fragment lengths was that both average specificity and average sensitivity dropped when oligonucleotides longer than 5 were analyzed. In Additional file 1 the oligonucleotide length-dependent classification accuracy is exemplified using sequence of length 800 bp and 50 Kbp. Conversely, the false negative rate increased when longer oligonucleotide lengths were considered (Additional file 1). A detailed table summarizing average accuracy values and standard deviations for the two different fragment lengths (800 bp and 50 Kbp) and for each oligonucleotide length analyzed is given as Additional file 2.

The kernel parameter *λ *governs the width of the local neighborhood, thus influencing the local behavior of the decision boundary allowing to search for an optimal trade-off between a well-fitted and a more generalized classifier.

A grid search (2 ≤ *λ *≤ 1000) was employed to detect values of *λ *resulting in maximal accuracy values (*λ*_**opt**_). In general, *λ*_**opt **_is smaller at lower taxonomic ranks (Table 1). This observation may be explained by the drastic increase on the number of taxonomic classes at deeper ranks. If a large number of taxonomic classes occur at deeper ranks, the neighborhood to be considered in the classification task needs to be smaller (small *λ*) than for broader taxonomic ranks. On the other hand, if a large *λ *is considered and a large number of classes exists, the respective neighborhood of a query genomic vector may cover too many reference vectors from diverse taxonomic classes; resulting in a negative impact on the classification accuracy. However, if the reference vectors from a taxonomic class are sparsely distributed from the query genomic vector, it is necessary to consider a bigger neighborhood (large *λ*). This may explain those cases where a large *λ*_**opt **_is obtained.

**Table 1:** Optimized parameter obtained for each genomic fragment length at each taxonomic rank

	*λ*_**opt**_				
	
**Fragment length**	**S**	**P**	**C**	**O**	**G**
800 bp	500	300	100	25	100

1 Kbp	500	300	200	100	100

3 Kbp	500	300	300	500	400

10 Kbp	300	400	300	100	90

15 Kbp	400	300	500	200	100

50 Kbp	500	1000	400	500	80

Optimal lambda parameter (*λ*_**opt**_) is shown for each genomic fragment length at each taxonomic rank: Superkingdom (S), Phylum (P), Class (C), Order (O), and Genus (G).

**Figure 2:**
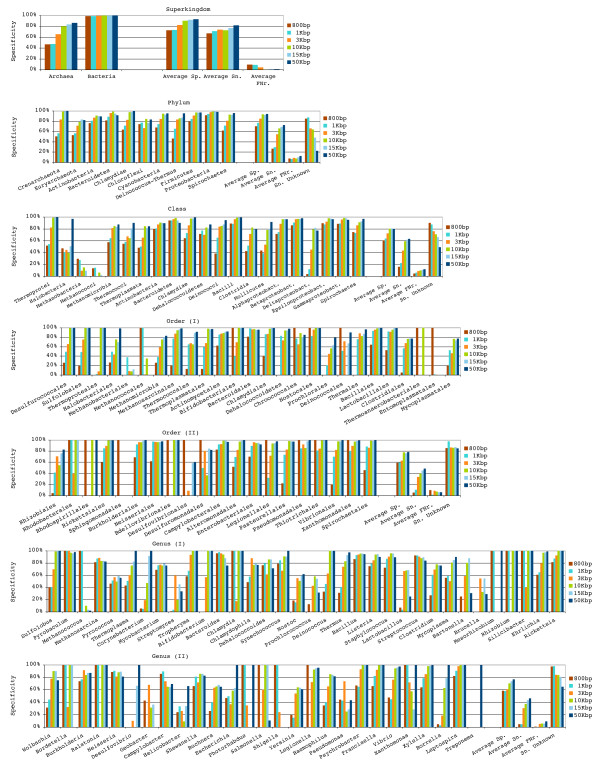
**Classification accuracy achieved for genomic fragments of different lengths**. Bars depict detailed specificity and average values for specificity (Sp.), sensitivity (Sn.) and false negative rate (FNr.) for each fragment length on different taxonomic ranks. Each color represents a genomic fragment length.

During the optimization procedure, optimal parameters were chosen based on average accuracy values over all taxonomic classes at each taxonomic rank, therefore it may occur that the optimal parameters chosen are indeed suboptimal for some taxonomic classes at a given rank. In consequence, the accuracy for some taxonomic classes can drop dramatically, this situation can be seen as "gaps" in Figure 2.

From a practical perspective we regarded it to be more valuable to produce a low number of highly reliable predictions rather than a large number of predictions with low reliability. Therefore, in this study we favored parameters that produce a high specificity rather than a high sensitivity.

### Classification accuracy for genomic fragments of variable length

The classification accuracy of TACOA was evaluated on genomic fragments of lengths ranging from 800 bp to 50 kbp. A total of 11,730,382 genomic fragments from 373 different species were analyzed, comprising ≈42 Mb of sequence data. The classification accuracy for all different evaluated genomic fragment lengths, taxonomic ranks, and taxonomic classes is given in detail in Figure 2.

**Figure 3:**
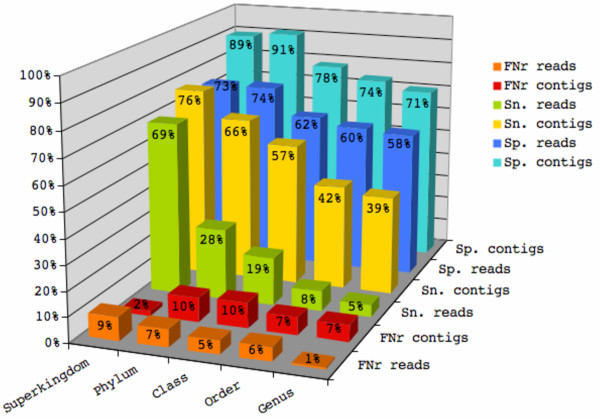
**Overall performance for reads and contigs for each taxonomic rank**. Average sensitivity (Sn.), specificity (Sp.), and false negative rate (FNr.) achieved for reads and contigs at each taxonomic rank.

A high proportion of contigs (genomic fragments of length 3 Kbp, 10 Kbp, 15 Kbp, and 50 Kbp) was correctly classified with an average sensitivity between 76% at rank superkingdom and 39% at rank genus (Figure 3). At the same time, less than 10% of contigs were misclassified (false negative rate) at all taxonomic ranks. For the remaining contigs the taxonomic origin could not be inferred and hence these were assigned to the "unclassified" class. Overall, reliable predictions were obtained with an average specificity ranging from 89% at superkingdom to 71% at rank genus. For the longest analyzed contig length (50 Kbp), TACOA achieved an average sensitivity of 82% at superkingdom and 46% at genus, and specificity of 93% (superkingdom) and 77% (genus) (Figure 2, Additional file 3). Also for shorter contigs, a high classification accuracy was obtained. For example, 74% of the contigs of length 3 Kbp were correctly classified at rank superkingdom and 31% at rank genus (Figure 2, Additional file 3), the specificity for contigs of length 3 kbp reached values between 74% (superkingdom) and 31% (genus).

In this evaluation, single reads were represented by genomic fragments of length 800 bp – 1 Kbp. TACOA is capable of accurately predicting the taxonomic origin of single reads up to the rank of class, despite the limited information contained in these short sequences. A high proportion of reads was correctly classified. For reads of length 800 bp, the average sensitivity was between 67% at superkingdom to 16% at rank class and for reads of length 1 Kbp, it ranged from 71% to 22%. Furthermore, in average only between 9% (superkingdom) and 5% (class) of reads were misclassified. Overall, reliable predictions were obtained, with an average specificity ranging from 73% (superkingdom) to 62% (class) for 800 bp reads and between 73% and 64% for reads of length 1 Kbp. In light of the limited information contained in fragments of length 800 bp – 1 Kbp and the complexity of the classification problem (e.g. 62 classes on rank genus), TACOA also achieves a surprisingly good performance for single reads at rank order and genus (Additional file 3).

However, in practice it is not recommended to interpret classification results of single reads on these ranks because only a small number of fragments may be represented in the currently available sequenced genomes. In real metagenomic data sets, already sequenced organisms may be contained in the studied sample. Therefore, the classification accuracy of TACOA was also assessed for fragments stemming from organisms included in the reference set (Additional file 4). As expected, having the source organisms of classified fragments included in the reference set has a markedly positive impact on the accuracy at all taxonomic ranks. The sensitivity increased of up to 30%. Furthermore, the specificity substantially increased while the false negative rate was reduced (Additional file 4).

As a general trend, the accuracy improves when longer genomic fragments were classified (Figure 2, Additional file 3). For example, on rank superkingdom the sensitivity increased from 67% for 800 bp reads to 82% for 50 Kbp contigs and at rank genus from 5% to 46%. Conversely, the accuracy decreases as deeper taxonomic ranks were examined (Figure 3, Additional file 3, Additional file 4). In general, it is easy to predict classes that are well represented in the reference set, while detecting the underrepresented taxonomic groups is more challenging (Figure 2). TACOA is capable of detecting a remarkably high number of different taxonomic classes, if they are present in a studied sample. For example for contigs of length 3 Kbp, TACOA achieved a sensitivity above 20% for all 11 phyla, for 18 of the 21 classes, for 30 of the 45 orders, and for 33 of the 61 genera represented in our test set (Additional file 5 and Additional file 6).

### Assessing the classification accuracy of TACOA and PhyloPythia for genomic fragments of variable length

We compared the classification accuracy (sensitivity, specificity and false negative rate) of our proposed kernelized *k*-NN classification method with PhyloPythia, which employs a hierarchical collection of SVMs for the taxonomic classification of environmental fragments. The set of completely sequenced genomes used for comparison was selected as follows: at rank class, two different genomes were randomly chosen from each taxonomic class guaranteeing that the data set used in the comparison is unbiased. Moreover, the genomes were randomly selected from the universe of all recently published genomes ensuring that the test set is not contained in the training set of PhyloPythia or reference set of TACOA. The selected test set resembles very well the situation when the classifiers need to predict the taxonomic origin of organisms that have not yet been sequenced.

**Figure 4:**
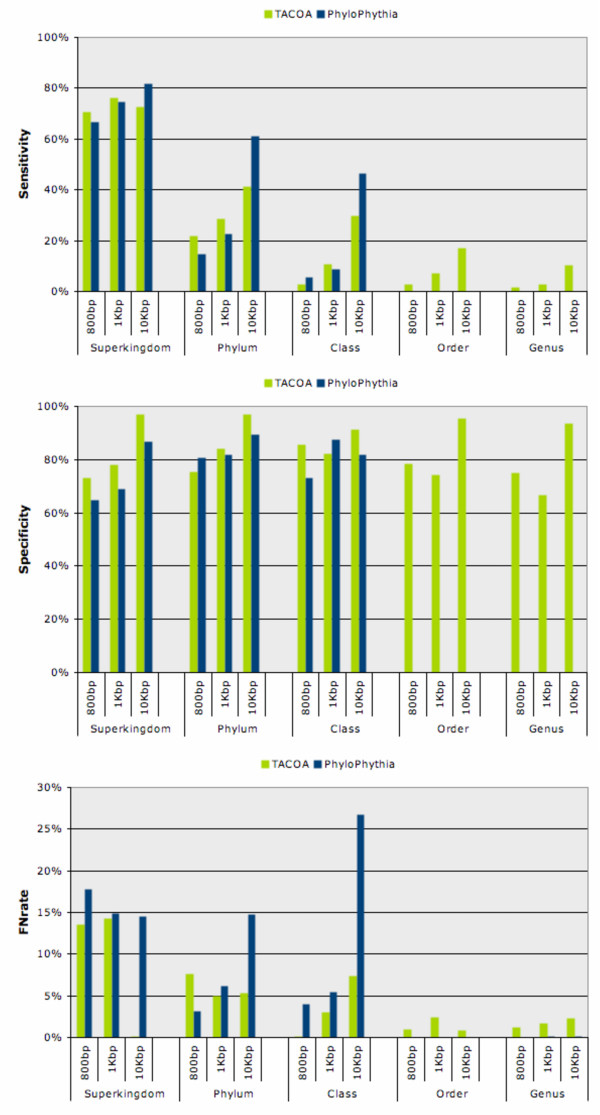
**Classification accuracy obtained for TACOA and PhyloPythia**. Sensitivity (top), specificity (middle) and false negative rate (bottom) achieved by TACOA and PhyloPythia for three different genomic fragment lengths and taxonomic ranks evaluated. Single read lengths are represented by fragments of length 800 bp and 1 Kbp and contigs by 10 Kbp long fragments. The accuracy achieved is depicted using green bars for TACOA and blue bars for PhyloPythia. The sensitivity and specificity charts are scaled between 0–100% and the false negative rate is scaled between 0–30%.

In general, TACOA and PhyloPythia achieved quite comparable classification accuracies, but TACOA has a slightly improved performance for the classification of short DNA fragments. For the classification of reads of length 800 bp and 1 Kbp, TACOA has a higher sensitivity while both tools achieve a comparable false negative rate and specificity values (Figure 4). Remarkably, on ranks order and genus TACOA is still able to correctly classify between 3% and 17% of short fragments (sensitivity), while PhyloPythia cannot infer the taxonomic origin of any of the fragments and thus has an average sensitivity of 0%. For longer contigs (DNA fragments of length 10 Kbp) PhyloPythia is more sensitive on higher taxonomic ranks (superkingdom, phylum and class). In contrast, TACOA produces less misclassifications (false negative rate) making its prediction more reliable. On lower taxonomic ranks (genus and order), TACOA is able to correctly infer the taxonomic origin of about 10% to 17% of all contigs, while PhyloPythia has a sensitivity of 0% for all taxonomic groups at these ranks.

A closer analysis of the classification of short DNA fragments, across ranks superkingdom to class, reveals that TACOA achieved sensitivity values of 71% to 3% for 800 bp fragments and 76% to 11% for 1 Kbp fragments. On the other hand, at ranks superkingdom, phylum and class PhyloPythia obtained a slightly lower sensitivity of 66% to 6% for 800 bp fragments and 75% to 9% for 1 Kbp fragments. At deeper ranks order and genus, TACOA is able to correctly classify between 3% and 7% of all short fragments (sensitivity), while only between 1% and 2.43% of fragments are misclassified (false negative rate). In contrast, PhyloPythia was not able to predict any taxonomic class resulting in a sensitivity of 0% for all groups on these two ranks. Overall, for short fragments TACOA is more sensitive at almost all taxonomic ranks, in particular at ranks order and genus. The only exception is at rank class, at which PhyloPythia is more sensitive for the classification of 800 bp fragments. At the same time, for the classification of short fragments TACOA has a slightly lower false negative rate for almost all taxonomic ranks. The only exceptions are rank phylum at which PhyloPythia has a lower false negative rate for 800 bp fragments. For the classification of contigs of length 10 Kbp, TACOA achieved a sensitivity between 73% and 30% at ranks superkingdom to class, while PhyloPythia correctly classified between 82% and 47%. According to these results PhyloPythia was between 9% and 17% more sensitive than TACOA. But for the same contig length and ranks, TACOA is between 10% and 9% more specific than PhyloPythia. In addition, a high percentage of misclassifications was also observed for PhyloPythia (18.64% in average) in contrast to that achieved by TACOA (4.30% in average). At lower taxonomic ranks, TACOA achieved average sensitivity values between 17% (order) and 10% (genus) for the classification of 10 Kbp contigs, while PhyloPythia was not able to predict any taxonomic class for these long contigs, thus obtaining a sensitivity of 0% (Figure 4). Although PhyloPythia was not able to make predictions for ranks order and genus, a marginal misclassification rate was observed (0.14% at rank order and 0.10% at rank genus) for a fragment length of 10 Kbp. Detailed sensitivity, specificity and false negative rate values for all taxonomic ranks and evaluated lengths are given in Additional file 7, Additional file 8 and Additional file 9.

### Influence of horizontal gene transfer on the classification accuracy of an intrinsic-based classifier

The classification accuracy of methods using composition-based features might be influenced by a heterogeneous nucleotide composition present in the DNA sequence of the analyzed genomic fragment. Although differences in the nucleotide composition of DNA sequences can be linked to a number of genomic attributes, including codon usage, DNA base-stacking energy, DNA structural conformation, strand asymmetry and even relic features of the primary genetic information, horizontal gene transfer events (HGT) is one of the most common cause [32,33]. The work of Brown *et al*. also suggests that despite the rapid changes on the nucleotide composition of recent transferred DNA chunks, the phylogenetic signal from the donor can still be detected if the HGT event is recent, rather than ancient [34]. Since the importance of HGT events has been gaining increasing attention lately [35], we investigated its influence in the accuracy of the intrinsic-based classifier TACOA.

One of the findings of this work is that tetranucleotides were best suited to analyzed genomic fragments ≤ 3 Kbp. But it has been reported that tetranucleotide frequencies are a good measure to detect horizontally transferred regions [36]. Therefore, any classifier aiming to predict the taxonomic origin of genomic fragments based on a tetranucleotide feature is susceptible to "wrongly" classify to the donor taxonomic class a genomic fragment obtained via HGT. To explore the influence of HGT events in the classification accuracy of TACOA, we selected fragments of length 1 Kbp from two genomes (one archaeal and one bacterial). Several studies [37-40] have reported acquisition of large stretches of DNA via HGT events for *Thermoplasma acidophilum *(archaea) and for *Thermotoga maritima *(bacteria).

In particular, the archaeal genome of *Thermoplasma acidophilum *has been reported to acquire ≈12% of its genome via HGT. The main donors seem to belong to bacterial organisms, but also some archaeal species have been detected [37,38]. It has been suggested that *T. acidophilum *has received genes via HGT from *Sulfolobus solfataricus*, a distantly related crenarchaeota living in the same ecological niche [38,39]. The sensitivity achieved by TACOA for *T. acidophilum *was 43% for reads 800 bp long and 51% for reads of length 1 Kbp.

**Figure 5:**
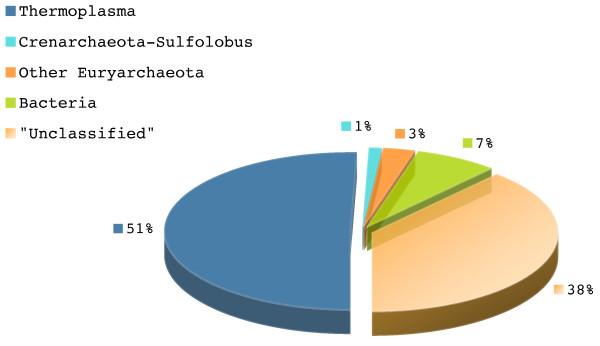
**Distribution of taxonomic assignments for Thermoplasma acidophilum**. Proportions of genomic fragments originating from the *T. acidophilum *genome that are misclassified into other taxonomic groups.

In order to evaluate the taxonomic distribution of misclassifications for *T. acidophilum *genomic fragments, we fragmented its genome in pieces of length 1 Kbp and predicted their taxonomic origin. For the 1,564 fragments analyzed, we found that 1% (16 from 1,564) were misclassified into the order sulfolobales, another 3% (47 from 1,564) into other members of the euryarchaeota group, 7% (110 from 1,564) to a variety of members from the bacterial group, and 38% (601 from 1,564) could not be classified (Figure 5). From the proportion of genomic fragments that were "erroneously" misclassified, the largest fraction (7%) was placed into the sulfolobus group. The results of the taxonomic distribution of "misclassifications" made by TACOA for *T. acidophilum *are in close agreement to previous studies [37,38]. Hence, the low number of correctly classified fragments obtained for *T. acidophilum *at rank genus may be partially explained by the lateral transfered DNA from other species.

We also explored the bacterial genome of *Thermotoga maritima*, which is another organism with a high number of candidate genes that have been presumably acquired from archaea via HGT [37]. A total of 1,860 genomic fragments of length 1 Kbp each were classified using TACOA and analyzed (Additional file 8). A high number of misclassified genomic fragments were "wrongly" assigned to the archaeal group (91 from 1,860), a small fraction (27 from 1,860) was erroneously assigned to the sulfolobus group and 27% (503 from 1,860) could not be classified. Conversely to *T. acidophilum*, the genome *T. maritima *seems to be recipient of DNA originating mainly from archaeal species as suggested by other authors [37-40]. These two case studies strongly suggest that horizontally transfered stretches of DNA can affect the classification accuracy of a classifier using compositional based features to infer the taxonomic origin of genomic fragments. A possible explanation for this observation is that the nucleotide composition of transferred DNA chunks still carry phylogenetic signals from the donor genome after the HGT event has occurred as suggested by Brown [34].

## Discussion and conclusion

Our novel strategy named TACOA can accurately predict the taxonomic origin of genomic fragments from metagenomic data sets by combining the advantages of the *k*-NN approach with a smoothing kernel function. The reference set used by our proposed method can be easily updated by simply adding the Genomic Feature Vectors (GFVs) from the new genomes to the reference set without the need of retraining. Our standalone tool TACOA can also be easily installed and run on a desktop computer, therefore allowing researchers to locally analyze their metagenomic sequence data or integrate it into their pipelines.

Analogous to PhyloPythia, researchers can easily incorporate sample specific-models from particular organisms into the framework of TACOA. The use of sample-especific models can greatly support the identification of organisms of special interest. Sample specific-models can be easily incorporated into the framework of TACOA by the researcher using the following approach: Genomic fragments carrying phylogenetic marker genes (such as rRNA genes) or fragments with high similarity to reference sequences of known origin (identified using a blast search) can be taxonomically annotated in a pre-processing step. Subsequently, these annotated fragments can be added to the reference set of TACOA. This can be easily done with the "addReferenceGenome" program provided by TACOA. The use of sample-specific models will improve the accuracy of the classifier for those species that have a reference sequence in public databases (i.e. because the test set is contained in the reference set). In this work, we demonstrated that having the test set in the reference set can have a positive impact on the sensitivity and specificity of up to 30% and at the same time a decline on the false negative rate is observed (Additional file 4).

As a whole, we evaluated the classification accuracy at five different taxonomic ranks: Superkingdom, Phylum, Class, Order, and Genus. TACOA can correctly classify genomic fragments of length as short as 800 bp up to rank class. Our proposed method can be used to predict the taxonomic origin of genomic fragments sequenced from any technology producing fragments ≥ 800 bp. Our strategy also produced reliable predictions for genomic fragments originating from taxonomic groups that are absent from the reference set (simulating fragments stemming from genomes not yet sequenced). On average and over all taxonomic ranks, 77% of these fragments were correctly classified as "unknown".

TACOA compares well to the current most sophisticated taxonomic classifier for environmental fragments PhyloPyhtia. In terms of percentage of correctly classified fragments (sensitivity) TACOA slightly outperforms PhyloPythia for reads of length 800 bp and 1 Kbp at all taxonomic ranks evaluated, except for reads 800 bp at rank class. But the very low false negative rate (0.16%) and the high specificity (86%) of TACOA makes the accuracy for reads of length 800 bp (at rank class) comparable to that obtained by PhyloPythia. Compared to TACOA, the overall reduced sensitivity obtained by PhyloPythia (evident for the analyzed read lengths) is partially due to the absence of the phylum Chloroflexi and Thermatogae from its training set. This example illustrates the positive effect of an updated training or reference set in the prediction of known taxonomic classes.

For contigs of length 10 Kbp, TACOA achieved lower sensitivity, lower false negative rate and higher specificity values than PhyloPyhtia. Although PhyloPythia achieves higher sensitivity values for contigs of length 10 Kbp the overall performance is comparable for both classifiers at ranks superkingdom, phylum and class.

At deeper taxonomic ranks (order and genus), for all evaluated lengths TACOA was still able to provide correct classifications for several taxonomic classes (average sensitivity of about 7%) while PhyloPythia failed in making any taxonomic assignments (sensitivity of 0%). With an average sensitivity of 17% (order) and 10% (genus), an average false negative rate of 1.45% (order) and 2.29% (genus), TACOA can provide a more detailed view of the taxonomic composition of an environmental sample. Notice that in practice it is not recommended to draw conclusions at such deep ranks for reads ≤ 1 Kbp because only a small number of fragments may be represented in the currently available sequenced genomes.

An interesting observation made during this work was that the classification of genomic fragments is possible using only GFVs computed from completely sequenced genomes rather than computing the vectors on fragments from genomes. Similar observations have already been made by Abe *et al*. in 2005 and 2006 and more recently by McHardy *et al*. in 2007, where the developed classifiers were trained with genomic fragments longer than the ones being tested. Here we demonstrated that even complete genomes can be used as reference to classify environmental genomic DNA fragments.

This study supports the findings that frequencies of short length oligonucleotides (i.e. tetra- and penta-oligonucleotides) are best suited to capture taxon-specific differences among prokaryotic genomes [10,11,16,20]. Moreover, our parameter search analysis strongly suggests that tetra- or penta-oligonucleotide frequencies are optimal features for TACOA to classify environmental genomic fragments as short as 800 bp. This observation is in accordance to those reported by Bohlin *et al*. [32] who already proposed that little increase in information potential about phylogenetic relationships is gained in oligonucleotide sizes larger than hexa-nucleotides.

We showed that recent events of HGT can affect the accuracy of a composition-based classifier. The correct classification of horizontally transferred regions into its "current" taxon is difficult if these still carry a strong phylogenetic signal from the donor genome. This was illustrated by classifying fragments of length 1 Kbp from the archaea *T. acidophilum *and the bacteria *T. maritima*. Notably, HGT is not the only phenomena causing variations in the oligonucleotide frequencies within genomes and hence affecting the classification performance.

TACOA combines the ability of predicting the taxonomic origin of genomic fragments with high accuracy and the advantage of being a tool that can easily be installed and used on a desktop computer breaking any dependency and limitations that web server services may bring. Altogether, it strongly suggests that TACOA offers a great potential to assist on the exploration of the taxonomic composition of metagenomic data sets.

## Methods

### Computation of genomic feature vectors (GFV) using the oligonucleotide frequency deviation

In the following, the computation of GFVs used by the TACOA classifier is described in detail. Computation of the GFVs is performed for each genome in the reference set and for each read and contig to be classified.

An oligonucleotide *o *is defined as a string over the alphabet ∑ = {*a*, *t*, *c*, *g*}. The total number of possible oligonucleotides of length *l *is given by 4^*l*^, e.g. for *l *= 3 oligonucleotides can take the form of *o*^[1] ^= *aaa*, *o*^[2] ^= *aat*, ..., *o*^[64] ^= *ggg*. To build a GFV for a genomic fragment, for each oligonucleotide the oligonucleotide deviation score is computed as the ratio between the observed oligonucleotide frequency in the fragment and the expected oligonucleotide frequency in that fragment given its GC-content. The GC-content has a profound impact on the sequence composition of genomes but a low phylogenetic signal. It has been shown that closely related organisms coming from different environments may show profound differences in GC-content [41].

More formally, given a genomic fragment *s*, for each oligonucleotide *o*^[*y*]^(*y *= 1, 2, 3, ..., 4^*l*^) we count the number of occurrences of *o*^[*y*] ^in *s*. The counting of the oligonucleotide frequencies is conducted in a sliding window approach with step size of 1 and window size *l*. This ratio is carried out on the forward and reverse DNA strand.

In order to more efficiently recover the phylogenetic signal contained in the oligonucleotide frequency deviation, we correct for biases introduced by the GC-content of the genomic fragments. The expected frequency for a certain oligonucleotide *o *in a genomic fragment *s *can be estimated by:

(5) $$E[o]\approx |s|\prod_{q=1}^{\left|o\right|}p(o_{q})$$

where *o*_*q *_is the nucleotide at position q of *o *and *p*(*o*_*q*_) defines the probability to observe *o*_*q *_in the analyzed genomic fragment, given its GC-content. The length of a genomic fragment is defined as |*s*| and |*o*| is the length of an oligonucleotide. Let *O*[*o*] be the observed occurrence of oligonucleotide *o *in the analyzed genomic fragment, then *p*(*o*_*q*_) is estimated by $p(o_{q})=\frac{O[o]}{\left|s|-(l-1\right)}$. For each oligonucleotide *o*, a deviation score *g*(*o*) is computed in a given genomic fragment, which is normalized by the GC-content. The deviation score *g*(*o*) resolves for under and over-represented oligonucleotide frequencies in a genomic fragment. The deviaton score *g*(*o*) is given by:

(6) $$g(o)=\{\begin{array}{ccc} 0 & \text{if} & O[o]=0\\ \frac{O[o]}{E[o]} & \text{if} & O[o]>E[o]\\ -\frac{E[o]}{O[o]} & \text{if} & O[o]\leq E[o] \end{array}$$

The computed *g*(*o*) for each possible *o*^[*y*] ^of length *l *in a given genomic fragment is summarized in a GFV **x **(Equation 7), this approach is also referred to as the vector representation model [29].

(7) $$x={\left(f(o^{\left[1\right]}),g(o^{\left[2\right]}),...,g(o^{\left[4^{l}\right]})\right)}^{T}$$

### Measuring the classification accuracy

We selected different genomic fragment lengths to simulate DNA fragments obtained in real metagenomic sequencing projects. Genomic fragments of length 800 bp and 1 Kbp were chosen to resemble single reads derived by the Sanger technology. Assembled contigs were simulated selecting fragment lengths of 3 Kbp, 10 Kbp, 15 Kbp, and 50 Kbp. Genomic fragment generation was executed in the following manner: For each completely sequenced genome and for each chosen genomic fragment length, 3000 non-overlapping fragments were extracted from the selected genome and subsequently included into the test set.

We estimated the classification accuracy of the presented method (TACOA) based on the leave-one-out cross-validation strategy. We selected one genome from the 373 different organisms, generated genomic fragments of a given length |*s*|, represented them as GFVs and predicted their taxonomic origin using the remaining 372 organisms as the reference set (**ref**_*set*_). Hereby, each of the 372 genomes in the reference set is represented as a GFV. This procedure was repeated for each genome out of the 373 completely sequenced genomes present in the data set (Figure 1).

The classification accuracy of the presented method was assessed at each taxonomic rank. At each taxonomic rank, the predicted class of each query genomic fragment was compared to its known taxonomic class. We evaluated the classification accuracy for those genomes having at least two different representatives per taxonomic class. Furthermore, we also evaluated the classification accuracy for those genomes only having one member per taxonomic class, in which case the method should assign them to the "unknown" class. The latter evaluation mimics the situation of organisms without a reference genome because they have not yet been sequenced. The classification accuracy of the presented method was assessed at each taxonomic rank.

In this study, we employed the adapted definition of sensitivity and specificity proposed by Baldi *et al*. in 2000 [42]. The classification accuracy was evaluated for each taxonomic class. Let the *i*-th taxonomic class of taxonomic rank *r *be denoted as class *i*. Further, let *Z*_*i *_be the total number of genomic fragments from class *i*, the true positives (*TP*_*i*_) the number of genomic fragments correctly assigned to class *i*, the false positives (*FP*_*i*_) the number of fragments from any class *j *≠ *i *that is wrongly assigned to *i*. The false negatives (*FN*_*i*_) is defined as the number of fragments from class *i *that is erroneously assigned to any other class *j *≠ *i*. For a genomic fragment whose taxonomic class cannot be inferred, the algorithm classifies it as "unclassified". The unclassified (*U*_*i*_) are the number of fragments from class *i *that cannot be assigned to a taxonomic class, so *Z*_*i *_= *TP*_*i *_+ *FN*_*i *_+ *U*_*i*_.

The sensitivity (**Sn**_*i*_) for a taxonomic class *i *is defined as the percentage of fragments from class *i *correctly classified and it is computed by:

(8) $$Sn_{i}=\frac{TP_{i}}{Z_{i}}$$

The reliability (expressed in percentage) of the predictions made by the classifier for class *i *is denoted as specificity (**Sp**_*i*_) and it is measured using the following equation:

(9) $$Sp_{i}=\frac{TP_{i}}{TP_{i}+FP_{i}}$$

Note that the specificity for class *i *is undefined for those cases when the terms *TP*_*i *_and *FP*_*i *_are both zero (marked as (-) in Additional figures 7 – 9). The overall specificity is computed over those classes that have a defined specificity value.

We make use of the false negative rate (**FNr**_*i*_) to measure the percentage of items from class *i *that is misclassified into any class *j *≠ *i*, which is given by:

(10) $$FNr_{i}=\frac{FN_{i}}{Z_{i}}$$

### Measuring the classification accuracy in the comparison of PhyloPythia and TACOA

The set of completely sequenced genomes used for comparison was selected as follows: at rank class, two different genomes were randomly chosen from each taxonomic class guaranteeing that the data set used in the comparison is unbiased. This procedure yielded a set of 63 genomes that were downloaded from the NCBI genome database [31]. For each evaluated fragment length and for each selected genome, ten non-overlapping genomic fragments were randomly extracted for classification. We evaluated both classification strategies at five different taxonomic ranks using three different genomic fragment lengths: 800 bp, 1 Kbp, and 10 Kbp. The PhyloPythia web server with the built-in generic model was employed to predict the taxonomic origin of genomic fragments generated from the 63 selected genomes. To predict the taxonomic origin of fragments from the same set of 63 selected genomes TACOA was executed using the default parameters. Notice that this evaluation aims to investigate the performance that a researcher should expect when analyzing their metagenomic data. The evaluation is not intended to assess the theoretical classification power of a kernelized *k*-NN against SVMs.

The accuracy of both classifiers was assessed using the sensitivity, false negative rate and specificity. Values of sensitivity, specificity and false negative rate were computed as previously described in this section. For the analysis of the comparison results between PhyloPythia and TACOA, we decided to give more emphasis to the obtained sensitivity and the false negative rates (FNr or misclassifications) to account for possible compositional biases of the data set. The sensitivity and the FNr measured for one class do not depend on the composition of the remaining classes (since the term false positive is absent in the equations of sensitivity and FNr). Hence, the sensitivity and FNr measured for each taxonomic group is not affected by possible biases of the test set. Contrastingly, the specificity measured for a class is strongly affected by the composition of the test set since it includes the false positives obtained from other classes.

## Availability

TACOA can be downloaded at 

## Authors' contributions

NND conceived, implemented, and performed the computational work, evaluated, and analyzed the data and drafted the manuscript. LK contributed to the implementation. KN and TWN supervised this work. AG provided the computational infrastructure for data generation and processing. All authors contributed to the editing of the manuscript.

## Supplementary Material

Additional file 1 **Oligonucleotide length-dependent performance for two different genomic fragment length.** Achieved specificity (left), sensitivity (middle) and false negative rate (right) for different oligonucleotide lengths in genomic fragments of length 800 bp (a) and 50 Kbp (b). For clarity the standard deviation was not depicted in these figures, instead is given as Additional file 2. Click here for file

Additional file 2 **Standard deviation for average accuracy and false negative rate achieved for different oligonucleotide lengths.** Standard deviation and average specificity, sensitivity and false negative rate is given for all oligonucleotide lengths and taxonomic ranks evaluated. Click here for file

Additional file 3 **Fragment-length and rank dependent performance.** Sensitivity (left) and specificity (right) achieved by TACOA for each genomic fragment length and taxonomic rank evaluated. Single read lengths are simulated by fragments 800 bp and 1 Kbp long and contigs by fragment lengths between 3 Kbp and 50 Kbp. Click here for file

Additional file 4 **Classification accuracy achieved using two different reference sets. **Each colored bar depicts the accuracy achieved by TACOA with two different reference sets. The label "Taxonomic organism of test fragment absent from reference set" refers when the test fragment is classified using a reference set not containing the source organism from which the test fragment originates from. Click here for file

Additional file 5 **Intervals for specificity (left) and sensitivity (right) of predicted taxonomic classes for reads.** Classification accuracy intervals for genomic fragments of length 800 bp (top) and 1 Kbp (bottom). Click here for file

Additional file 6 **Intervals for specificity (left) and sensitivity (right) of predicted taxonomic classes for contigs.** Classification accuracy intervals for genomic fragments of length 3 Kbp, 10 Kbp, 15 Kbp, and 50 Kbp (from top to bottom). Click here for file

Additional file 7 **Detailed accuracy obtained for genomic fragments of length 800 bp using TACOA and PhyloPythia classifiers.** At each taxonomic rank, the classification accuracy (specificity and sensitivity) achieved for two different intrinsic classifiers: TACOA and PhyloPythia is given. The symbol (-) refers to the cases where the respective value cannot be mathematically defined. Click here for file

Additional file 8 **Detailed accuracy obtained for genomic fragments of length 1 Kbp using TACOA and PhyloPythia classifiers.** At each taxonomic rank, the classification accuracy (specificity and sensitivity) achieved for two different intrinsic classifiers: TACOA and PhyloPythia is given. The symbol (-) refers to the cases where the respective value cannot be mathematically defined. Click here for file

Additional file 9 **Detailed accuracy obtained for genomic fragments of length 10 Kbp using TACOA and PhyloPythia classifiers. **At each taxonomic rank, the classification accuracy (specificity and sensitivity) achieved for two different intrinsic classifiers: TACOA and PhyloPythia is given. The symbol (-) refers to the cases where the respective value cannot be mathematically defined. Click here for file
