# Supplementary material for: TACOA – Taxonomic classification of environmental genomic fragments using a kernelized nearest neighbor approach
Source: BMC Bioinformatics. 2009 Feb 11;10:56. doi: 10.1186/1471-2105-10-56 (PMC2653487; doi:10.1186/1471-2105-10-56)
Supplement: Additional file 1 — Oligonucleotide length-dependent performance for two different genomic fragment length. Achieved specificity (left), sensitivity (middle) and false negative rate (right) for different oligonucleotide lengths in genomic fragments of length 800 bp (a) and 50 Kbp (b). For clarity the standard deviation was not depicted in these figures, instead is given as Additional file 2. [file 1471-2105-10-56-S1.pdf]

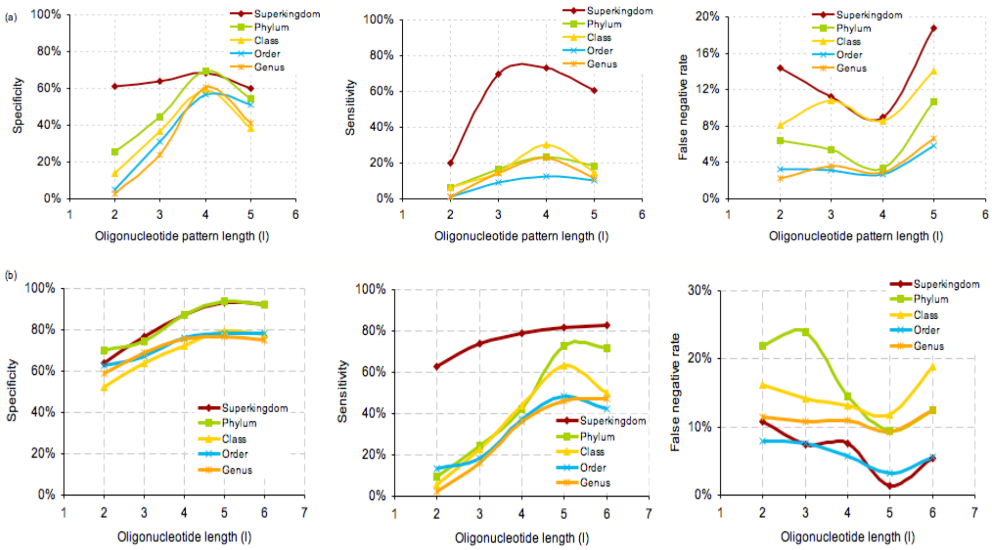

Additional figure 1: **Oligonucleotide length-dependent performance for two different genomic fragment length**. Achieved specificity (left), sensitivity (middle) and false negative rate (right) for different oligonucleotide lengths for genomic fragments of length 800bp (a) and 50Kbp (b). For clarity the standard deviation was not depicted in these figures, instead is given as Additional file 2.
