# Supplementary material for: TACOA – Taxonomic classification of environmental genomic fragments using a kernelized nearest neighbor approach
Source: BMC Bioinformatics. 2009 Feb 11;10:56. doi: 10.1186/1471-2105-10-56 (PMC2653487; doi:10.1186/1471-2105-10-56)
Supplement: Additional file 2 — Standard deviation for average accuracy and false negative rate achieved for different oligonucleotide lengths. Standard deviation and average specificity, sensitivity and false negative rate is given for all oligonucleotide lengths and taxonomic ranks evaluated. [file 1471-2105-10-56-S2.pdf]

Average specificty

Genomic fragment length (s)	Oligonucleotide length (l)	Superkingdom	σ ±	Phylum	σ ±	Class	σ ±	Order	σ ±	Genus	σ ±
800bp	2	0,61	0,51	0,26	0,28	0,14	0,19	0,05	0,16	0,03	0,12
	3	0,64	0,50	0,45	0,21	0,37	0,23	0,31	0,26	0,24	0,22
	4	0,73	0,44	0,70	0,23	0,61	0,28	0,59	0,28	0,58	0,24
	5*										
	6*										
50Kbp	2	0,64	0,50	0,70	0,19	0,52	0,35	0,63	0,46	0,59	0,38
	3	0,76	0,32	0,74	0,08	0,64	0,28	0,67	0,40	0,69	0,39
	4	0,87	0,17	0,87	0,05	0,72	0,22	0,76	0,30	0,75	0,27
	5	0,93	0,12	0,94	0,02	0,80	0,22	0,78	0,37	0,77	0,12
	6	0,92	0,24	0,92	0,02	0,78	0,05	0,79	0,29	0,75	0,28

Average sensitivity

Genomic fragment length (s)	Oligonucleotide length (l)	Superkingdom	σ ±	Phylum	σ ±	Class	σ ±	Order	σ ±	Genus	σ ±
800bp	2	0,20	0,52	0,26	0,20	0,06	0,10	0,05	0,26	0,03	0,01
	3	0,70	0,02	0,45	0,15	0,15	0,15	0,31	0,05	0,24	0,08
	4	0,73	0,07	0,69	0,15	0,30	0,12	0,57	0,20	0,60	0,09
	5*										
	6*										
50Kbp	2	0,63	0,12	0,09	0,18	0,06	0,16	0,13	0,12	0,02	0,11
	3	0,74	0,14	0,25	0,28	0,23	0,23	0,18	0,25	0,16	0,26
	4	0,79	0,11	0,42	0,28	0,44	0,29	0,37	0,33	0,36	0,36
	5	0,82	0,09	0,73	0,25	0,63	0,28	0,49	0,34	0,46	0,40
	6	0,83	0,05	0,72	0,31	0,50	0,29	0,42	0,34	0,47	0,41

Average false negative rate

Genomic fragment length (s)	Oligonucleotide length (l)	Superkingdom	σ ±	Phylum	σ ±	Class	σ ±	Order	σ ±	Genus	σ ±
800bp	2	0,1440	0,0220	0,0640	0,0360	0,0807	0,0287	0,0324	0,0665	0,0220	0,0247
	3	0,1126	0,0003	0,0543	0,0106	0,1074	0,0669	0,0319	0,0160	0,0360	0,0425
	4	0,0902	0,0001	0,0336	0,0032	0,0855	0,0767	0,0267	0,0120	0,0287	0,0357
	5*										
	6*										
50Kbp	2	0,11	0,03	0,22	0,00	0,16	0,02	0,02	0,03	0,08	0,06
	3	0,07	0,06	0,24	0,01	0,14	0,03	0,02	0,03	0,08	0,02
	4	0,08	0,09	0,15	0,01	0,13	0,03	0,02	0,04	0,06	0,03
	5	0,01	0,06	0,09	0,00	0,12	0,04	0,03	0,06	0,03	0,04
	6	0,05	0,04	0,12	0,01	0,19	0,01	0,05	0,12	0,05	0,09

*Oligonucleotide length not evaluated due to lack of minimal fragment length require to contain all posible oligonucleotide patterns

Additional Table 1: **Standard deviation for average accuracy and false negative rate achieved for fragments of length 800bp and 50Kbp using different oligonucleotide length**. Standard deviation and average specificity, sensitivity and false negative rate is given for all oligonucleotide lengths and taxonomic ranks evaluated.
