# Supplementary material for: TACOA – Taxonomic classification of environmental genomic fragments using a kernelized nearest neighbor approach
Source: BMC Bioinformatics. 2009 Feb 11;10:56. doi: 10.1186/1471-2105-10-56 (PMC2653487; doi:10.1186/1471-2105-10-56)
Supplement: Additional file 3 — Fragment-length and rank dependent performance. Sensitivity (left) and specificity (right) achieved by TACOA for each genomic fragment length and taxonomic rank evaluated. Single read lengths are simulated by fragments 800 bp and 1 Kbp long and contigs by fragment lengths between 3 Kbp and 50 Kbp. [file 1471-2105-10-56-S3.pdf]

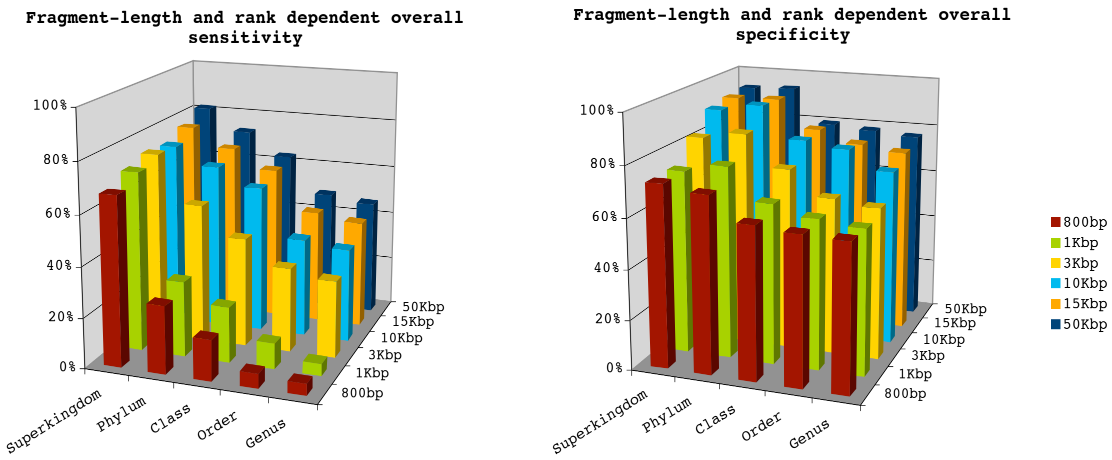

Additional figure 3: **Fragment-length and rank dependent performance.** Sensitivity (left) and specificity (right) achieved by TACOA for each genomic fragment length and taxonomic rank evaluated. Single read lengths are simulated by fragments 800bp and 1Kbp long and contigs by fragment lengths between 3Kbp and 50Kbp.
