# Supplementary material for: TACOA – Taxonomic classification of environmental genomic fragments using a kernelized nearest neighbor approach
Source: BMC Bioinformatics. 2009 Feb 11;10:56. doi: 10.1186/1471-2105-10-56 (PMC2653487; doi:10.1186/1471-2105-10-56)
Supplement: Additional file 4 — Classification accuracy achieved using two different reference sets. Each colored bar depicts the accuracy achieved by TACOA with two different reference sets. The label "Taxonomic organism of test fragment absent from reference set" refers when the test fragment is classified using a reference set not containing the source organism from which the test fragment originates from. [file 1471-2105-10-56-S4.pdf]

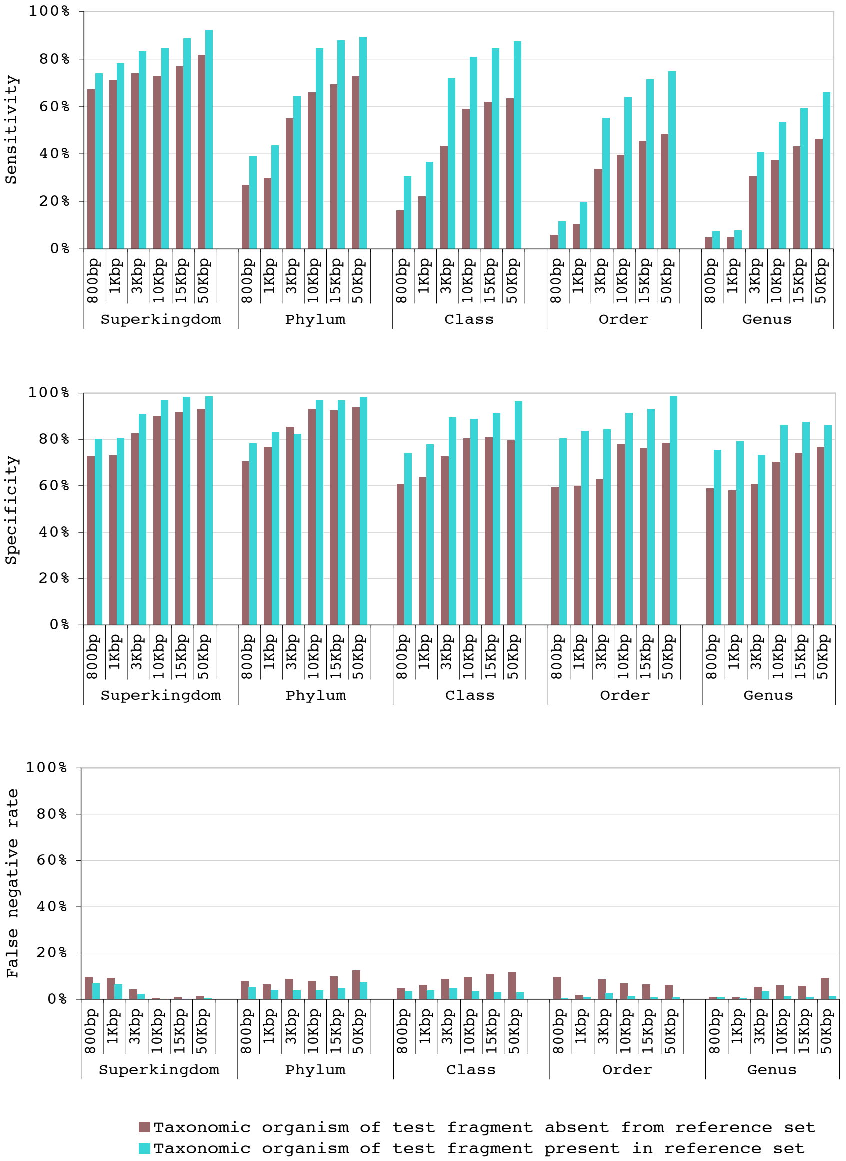

Additional figure 4: **Classification accuracy achieved using two different reference sets.** Each colored bar depicts the accuracy achieved by TACOA with two different reference sets. The label "Taxonomic organism of test fragment absent from reference set" refers when the test fragment is classified using a reference set not containing the source organism from which the test fragment originates from.
