# Supplementary material for: TACOA – Taxonomic classification of environmental genomic fragments using a kernelized nearest neighbor approach
Source: BMC Bioinformatics. 2009 Feb 11;10:56. doi: 10.1186/1471-2105-10-56 (PMC2653487; doi:10.1186/1471-2105-10-56)
Supplement: Additional file 5 — Intervals for specificity (left) and sensitivity (right) of predicted taxonomic classes for reads. Classification accuracy intervals for genomic fragments of length 800 bp (top) and 1 Kbp (bottom). [file 1471-2105-10-56-S5.pdf]

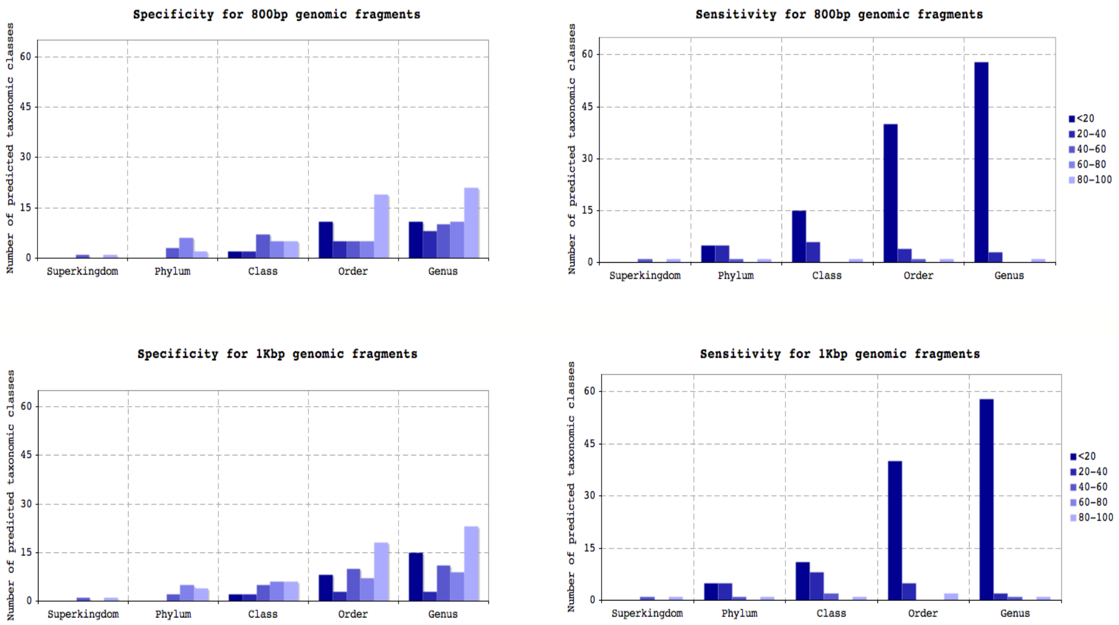


Additional figure 5: **Intervals for specificity (left) and sensitivity (right) of predicted taxonomic classes for reads.** Classification accuracy intervals for genomic fragments of length 800bp (top) and 1Kbp (bottom). The distribution of number of predicted taxonomic classes at each interval and per taxonomic rank is shown.
