# Supplementary material for: TACOA – Taxonomic classification of environmental genomic fragments using a kernelized nearest neighbor approach
Source: BMC Bioinformatics. 2009 Feb 11;10:56. doi: 10.1186/1471-2105-10-56 (PMC2653487; doi:10.1186/1471-2105-10-56)
Supplement: Additional file 6 — Intervals for specificity (left) and sensitivity (right) of predicted taxonomic classes for contigs. Classification accuracy intervals for genomic fragments of length 3 Kbp, 10 Kbp, 15 Kbp, and 50 Kbp (from top to bottom). [file 1471-2105-10-56-S6.pdf]

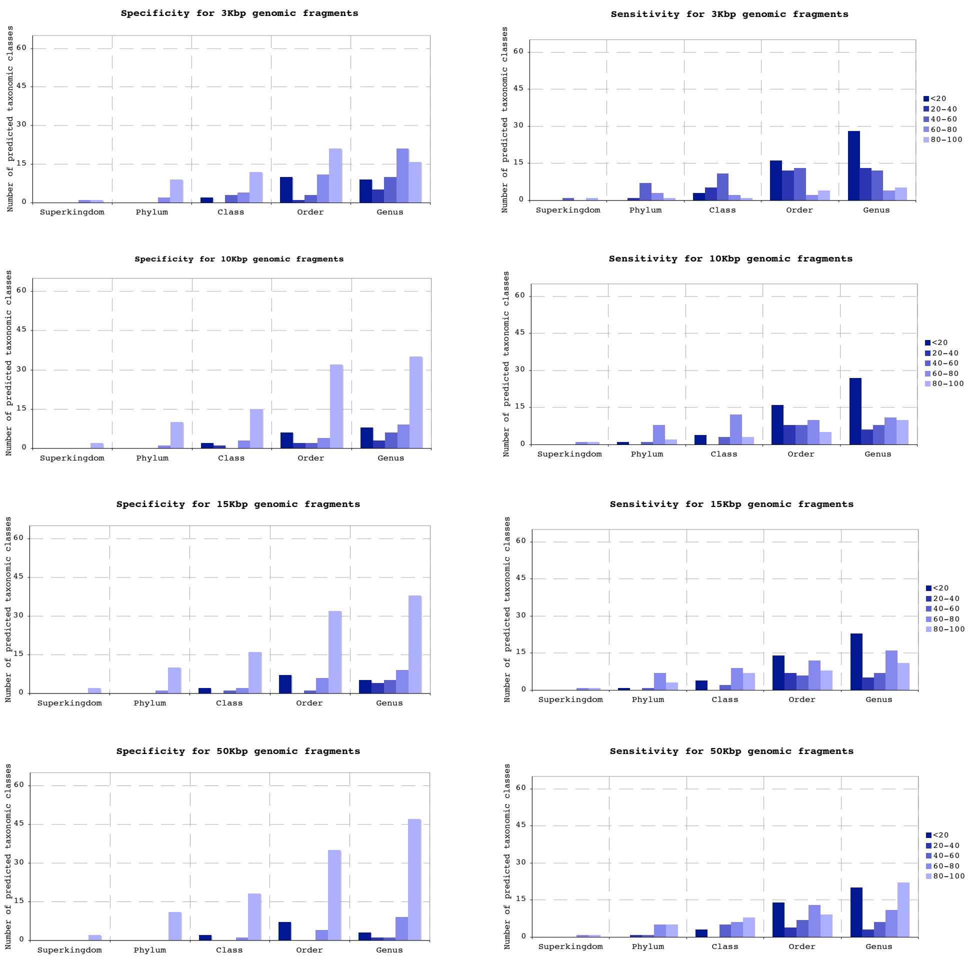

Additional figure 6: **Intervals for specificity (left) and sensitivity (right) of predicted taxonomic classes for contigs.** Classification accuracy intervals for genomic fragments of length 3Kbp, 10Kbp, 15Kbp, and 50Kbp (from top to bottom). The distribution of number of predicted taxonomic classes at each interval and per taxonomic rank is shown.
