# Supplementary material for: TACOA – Taxonomic classification of environmental genomic fragments using a kernelized nearest neighbor approach
Source: BMC Bioinformatics. 2009 Feb 11;10:56. doi: 10.1186/1471-2105-10-56 (PMC2653487; doi:10.1186/1471-2105-10-56)
Supplement: Additional file 7 — Detailed accuracy obtained for genomic fragments of length 800 bp using TACOA and PhyloPythia classifiers. At each taxonomic rank, the classification accuracy (specificity and sensitivity) achieved for two different intrinsic classifiers: TACOA and PhyloPythia is given. The symbol (-) refers to the cases where the respective value cannot be mathematically defined. [file 1471-2105-10-56-S7.pdf]

Additional file 7. Detailed accuracy obtained for reads 800bp long using TACOA and PhyloPythia							
		**Sensitivity**		**False Negative rate**		**Specificity**	
		**TACOA**	**PhyloPhythia**	**TACOA**	**PhyloPythia**	**TACOA**	**PhyloPhythia**
Superkingdom	Archaea	60%	55%	22,50%	27,50%	47%	32%
	Bacteria	81%	78%	4,66%	7,97%	98%	98%
**Average**		**71%**	**66%**	**13,58%**	**17,73%**	**73%**	**65%**
Phylum	Crenarchaeota	33%	0%	10,00%	6,67%	77%	-
	Euryarchaeota	40%	40%	0,00%	0,00%	36%	33%
	Actinobacteria	15%	55%	10,00%	0,00%	75%	100%
	Bacteroidetes	0%	35%	15,00%	0,00%	-	88%
	Chlamydiae	10%	0%	10,00%	10,00%	40%	-
	Chlorobi	0%	0%	20,00%	0,00%	-	-
	Chloroflexi	35%	0%	10,00%	0,00%	100%	-
	Cyanobacteria	5%	15%	2,50%	2,50%	100%	86%
	Firmicutes	11%	15%	6,25%	7,50%	57%	80%
	Proteobacteria	32%	16%	2,81%	0,61%	93%	98%
	Spirochaetes	45%	0%	0,00%	0,00%	75%	-
	Thermotogae	35%	0%	5,00%	10,00%	100%	-
**Average**		**22%**	**15%**	**7,63%**	**3,11%**	**75%**	**81%**
Class	Thermoprotei	17%	0%	3,33%	0,00%	100%	-
	Halobacteria	10%	0%	0,00%	0,00%	100%	-
	Actinobacteria	0%	0%	0,00%	0,00%	-	-
	Bacteroidetes	0%	10%	0,00%	0,00%	-	67%
	Chlamydiae	0%	0%	0,00%	0,00%	0%	-
	Chlorobia	0%	0%	0,00%	0,00%	-	-
	Chloroflexi	0%	0%	0,00%	0,00%	-	-
	Dehalococcoidetes	0%	0%	0,00%	0,00%	-	-
	Bacillales	0%	0%	0,00%	0,00%	-	-
	Bacilli	0%	0%	0,00%	10,00%	-	-
	Clostridia	0%	0%	0,00%	2,50%	-	-
	Alphaproteobacteria	7%	2%	0,00%	0,00%	100%	-
	Betaproteobacteria	2%	25%	0,00%	0,00%	100%	100%

	Burkholderiales	0%	0%	0,00%	60,00%	-	53%
	Deltaproteobacteria	0%	0%	0,00%	2,50%	-	-
	Epsilonproteobacteria	0%	80%	0,00%	0,00%	-	-
	Gammaproteobacteria	3%	0%	0,00%	4,62%	100%	73%
	Pasteurellales	0%	0%	0,00%	0,00%	-	-
	Rhodocyclales	0%	0%	0,00%	5,00%	-	-
	Spirochaetes	20%	0%	0,00%	0,00%	100%	-
	Thermotogae	0%	0%	0,00%	0,00%	-	-
**Average**		**3%**	**6%**	**0,16%**	**4%**	**86%**	**73%**

(-) Undefined value

		Sensitivity		False Negative rate		Specificity	
		TACOA	PhyloPhythia	TACOA	PhyloPythia	TACOA	PhyloPhythia
Order	Nitrosopumilales	0%	0%	0,10%	0,00%	-	-
	Sulfolobales	0%	0%	0,00%	0,00%	-	-
	Thermoproteales	20%	0%	30,00%	0,00%	100%	-
	Halobacteriales	10%	0%	0,00%	0,00%	100%	-
	Actinomycetales	0%	0%	0,00%	0,00%	-	-
	Bacteroidales	0%	0%	0,00%	0,00%	-	-
	Chlamydiales	0%	0%	0,00%	0,00%	-	-
	Chlorobiales	0%	0%	0,00%	0,00%	-	-
	Chloroflexales	0%	0%	0,00%	0,00%	-	-
	Dehalococcoidetes	0%	0%	0,00%	0,00%	-	-
	Chroococcales	0%	0%	0,00%	0,00%	-	-
	Prochlorales	0%	0%	0,00%	0,00%	-	-
	Bacilli	0%	0%	0,00%	0,00%	-	-
	Lactobacillales	0%	0%	0,00%	0,00%	-	-
	Clostridiales	0%	0%	0,00%	0,00%	-	-
	Thermoanaerobacteriales	0%	0%	0,00%	0,00%	-	-
	Rhodospirillales	0%	0%	0,00%	0,00%	-	-
	Rickettsiales	20%	0%	0,00%	0,00%	100%	-
	Sphingomonadales	0%	0%	0,00%	0,00%	-	-

	Burkholderiales	3%	0%	0,00%	0,00%	100%	-
	Nitrosomonadales	0%	0%	0,00%	0,00%	-	-
	Betaproteobacteria	0%	0%	0,00%	0,00%	-	-
	Desulfovibrionales	0%	0%	0,00%	0,00%	-	-
	Desulfuromonadales	0%	0%	0,00%	0,00%	-	-
	Campylobacterales	0%	0%	0,00%	0,00%	-	-
	Alteromonadales	5%	0%	0,00%	0,00%	50%	-
	Enterobacteriales	0%	0%	0,00%	0,00%	-	-
	Pasteurellales	0%	0%	0,00%	0,00%	-	-
	Pseudomonadales	0%	0%	5,00%	0,00%	-	-
	Thiotrichales	0%	0%	0,00%	0,00%	-	-
	Vibrionales	0%	0%	0,00%	0,00%	-	-
	Xanthomonadales	0%	0%	0,00%	0,00%	-	-
	Gammaproteobacteria	20%	0%	0,00%	0,00%	1%	-
	Spirochaetales	20%	0%	0,00%	0,00%	100%	-
	Thermotogales	0%	0%	0,00%	0,00%	-	-
**Average**		**3%**	**0%**	**1,00%**	**0%**	**79%**	**-**

(-) Undefined value

		Sensitivity		False Negative rate		Specificity	
		**TACOA**	**PhyloPhythia**	**TACOA**	**PhyloPythia**	**TACOA**	**PhyloPhythia**
Genus	Nitrosopumilus	0%		10,00%		-	-
	Metallosphaera	0%	0%	0,00%	0,00%	-	-
	Thermoproteus	0%	0%	40,00%	0,00%	-	-
	Halobacterium	10%	0%	0,00%	0,00%	-	-
	Mycobacterium	0%	0%	0,00%	0,00%	-	-
	Parabacteroides	0%	0%	0,00%	0,00%	-	-
	Porphyromonas	0%	0%	0,00%	0,00%	-	-
	Chlamydophila	0%	0%	0,00%	0,00%	-	-
	Chlamydia	0%	0%	0,00%	0,00%	-	-
	Chlorobium	0%	0%	0,00%	0,00%	-	-
	Chloroflexus	0%	0%	0,00%	0,00%	-	-

Dehalococcoides	0%	0%	0,00%	0,00%	-	-
Synechococcus	0%	0%	0,00%	0,00%	-	-
Prochlorococcus	0%	0%	0,00%	0,00%	-	-
Bacillus	0%	0%	0,00%	0,00%	-	-
Lactobacillus	0%	0%	0,00%	0,00%	-	-
Streptococcus	0%	0%	0,00%	0,00%	-	-
Clostridium	0%	0%	0,00%	0,00%	-	-
Thermoanaerobacter	0%	0%	0,00%	0,00%	-	-
Magnetospirillum	0%	0%	0,00%	0,00%	-	-
Ehrlichia	10%	0%	0,00%	0,00%	100%	-
Rickettsia	30%	0%	0,00%	0,00%	100%	-
Erythrobacter	0%	0%	0,00%	0,00%	-	-
Sphingomonas	0%	0%	0,00%	0,00%	-	-
Sphingopyxis	0%	0%	0,00%	0,00%	-	-
Ralstonia	0%	0%	0,00%	0,00%	-	-
Nitrosomonas	0%	0%	0,00%	0,00%	-	-
Nitrosospira	0%	0%	0,00%	0,00%	-	-
Burkholderia	10%	0%	0,00%	0,00%	100%	-
Desulfovibrio	0%	0%	0,00%	0,00%	-	-
Lawsonia	0%	0%	0,00%	0,00%	-	-
Geobacter	0%	0%	0,00%	0,00%	-	-
Campylobacter	0%	0%	0,00%	0,00%	-	-
Helicobacter	0%	0%	0,00%	0,00%	-	-
Shewanella	5%	0%	0,00%	0,00%	50%	-
Serratia	0%	0%	0,00%	0,00%	-	-

(-) Undefined value

		Sensitivity		False Negative rate		Specificity	
		**TACOA**	**PhyloPhythia**	**TACOA**	**PhyloPythia**	**TACOA**	**PhyloPhythia**
Genus (Cont.)	Shigella	0%	0%	0,00%	0,00%	-	-
	Acinetobacter	0%	0%	0,00%	0,00%	-	-
	Psychrobacter	0%	0%	10,00%	0,00%	-	-
	Francisella	0%	0%	0,00%	0,00%	-	-
	Thiomicrospira	0%	0%	0,00%	0,00%	-	-
	Vibrio	0%	0%	0,00%	0,00%	-	-
	Xanthomonas	0%	0%	0,00%	0,00%	-	-
	Xylella	0%	0%	0,00%	0,00%	-	-
	Actinobacillus	0%	0%	0,00%	0,00%	-	-
	Azoarcus	0%	0%	0,00%	0,00%	-	-
	Leptospira	20%	0%	0,00%	0,00%	100%	-
	Thermotoga	0%	0%	0,00%	0,00%	-	-
**Average**		**2%**	**0%**	**1,25%**	**0%**	**92%**	**-**

(-) Undefined value
