# Supplementary material for: TACOA – Taxonomic classification of environmental genomic fragments using a kernelized nearest neighbor approach
Source: BMC Bioinformatics. 2009 Feb 11;10:56. doi: 10.1186/1471-2105-10-56 (PMC2653487; doi:10.1186/1471-2105-10-56)
Supplement: Additional file 8 — Detailed accuracy obtained for genomic fragments of length 1 Kbp using TACOA and PhyloPythia classifiers. At each taxonomic rank, the classification accuracy (specificity and sensitivity) achieved for two different intrinsic classifiers: TACOA and PhyloPythia is given. The symbol (-) refers to the cases where the respective value cannot be mathematically defined. [file 1471-2105-10-56-S8.pdf]

Additional file 8. Detailed accuracy obtained for reads 1Kbp long using TACOA and PhyloPythia

| | | Sensitivity | | False Negative rate | | Specificity | |
|---|---|---|---|---|---|---|---|
| | | **TACOA** | **PhyloPhythia** | **TACOA** | **PhyloPythia** | **TACOA** | **yloPhythia** |
| Superkingdom | Archaea | 70% | 62% | 25,00% | 22,00% | 58% | 40% |
| | Bacteria | 82% | 88% | 3,45% | 7,83% | 98% | 98% |
| **Average** | | **76%** | **75%** | **14,22%** | **14,92%** | **78%** | **69%** |
| | | | | | | | |
| Phylum | Crenarchaeota | 40% | 3% | 6,67% | 12,50% | 92% | 100% |
| | Euryarchaeota | 70% | 50% | 0,00% | 10,00% | 64% | 28% |
| | Actinobacteria | 15% | 75% | 0,00% | 0,00% | 50% | 75% |
| | Bacteroidetes | 5% | 47% | 25,00% | 0,00% | 100% | 93% |
| | Chlamydiae | 40% | 0% | 0,00% | 10,00% | 89% | - |
| | Chlorobi | 0% | 0% | 5,00% | 5,00% | - | - |
| | Chloroflexi | 30% | 0% | 10,00% | 5,00% | 100% | - |
| | Cyanobacteria | 25% | 25% | 2,50% | 7,50% | 91% | 91% |
| | Firmicutes | 8% | 26% | 3,75% | 11,25% | 50% | 72% |
| | Proteobacteria | 33% | 34% | 1,88% | 3,03% | 96% | 97% |
| | Spirochaetes | 55% | 10% | 0,00% | 5,00% | 92% | 100% |
| | Thermotogae | 20% | 0% | 5,00% | 5,00% | 100% | - |
| **Average** | | **28%** | **22%** | **4,98%** | **6%** | **84%** | **82%** |
| | | | | | | | |
| Class | Thermoprotei | 27% | 0% | 3,33% | 0,00% | 100% | - |
| | Halobacteria | 40% | 0% | 10,00% | 10,00% | 100% | - |
| | Actinobacteria | 0% | 15% | 0,00% | 0,00% | 0% | 75% |
| | Bacteroidetes | 0% | 13% | 0,00% | 0,00% | - | 100% |
| | Chlamydiae | 30% | 0% | 0,00% | 0,00% | 38% | - |
| | Chlorobia | 0% | 0% | 5,00% | 0,00% | - | - |
| | Chloroflexi | 0% | 0% | 10,00% | 10,00% | - | - |
| | Dehalococcoidetes | 40% | 0% | 0,00% | 0,00% | 100% | - |
| | Bacillales | 0% | 0% | 0,00% | 5,00% | - | - |
| | Bacilli | 0% | 0% | 5,00% | 0,00% | - | - |
| | Clostridia | 3% | 0% | 0,00% | 0,00% | 100% | - |
| | Alphaproteobacteria | 2% | 5% | 3,33% | 0,00% | 100% | 100% |
| | Betaproteobacteria | 12% | 48% | 0,00% | 0,00% | 75% | 61% |

| | | | | | | | |
|---|---|---|---|---|---|---|---|
| | Burkholderiales | 0% | 0% | 0,00% | 70,00% | - | - |
| | Deltaproteobacteria | 0% | 0% | 5,00% | 0,00% | - | - |
| | Epsilonproteobacteria | 5% | 95% | 10,00% | 5,00% | 100% | 76% |
| | Gammaproteobacteria | 16% | 6% | 0,83% | 4,62% | 76% | 100% |
| | Pasteurellales | 0% | 0% | 0,00% | 0,00% | - | - |
| | Rhodocyclales | 0% | 0% | 10,00% | 10,00% | - | - |
| | Spirochaetes | 35% | 10% | 0,00% | 0,00% | 100% | 100% |
| | Thermotogae | 20% | 0% | 0,00% | 0,00% | 100% | - |
| **Average** | | **11%** | **9%** | **2,98%** | **5%** | **82%** | **87%** |

(-) Undefined value

| | | Sensitivity | | False Negative rate | | Specificity | |
|---|---|---|---|---|---|---|---|
| | | **TACOA** | **PhyloPhythia** | **TACOA** | **PhyloPythia** | **TACOA** | **yloPhythia** |
| Order | Nitrosopumilales | 0% | 0% | 10,00% | 0,00% | - | - |
| | Sulfolobales | 0% | 0% | 0,00% | 0,00% | 0% | - |
| | Thermoproteales | 40% | 0% | 20,00% | 0,00% | 100% | - |
| | Halobacteriales | 40% | 0% | 10,00% | 0,00% | 100% | - |
| | Actinomycetales | 0% | 0% | 0,00% | 0,00% | 0% | - |
| | Bacteroidales | 0% | 0% | 0,00% | 0,00% | - | - |
| | Chlamydiales | 30% | 0% | 0,00% | 0,00% | 38% | - |
| | Chlorobiales | 0% | 0% | 0,00% | 0,00% | - | - |
| | Chloroflexales | 0% | 0% | 10,00% | 0,00% | - | - |
| | Dehalococcoidetes | 0% | 0% | 0,00% | 0,00% | - | - |
| | Chroococcales | 0% | 0% | 0,00% | 0,00% | - | - |
| | Prochlorales | 0% | 0% | 0,00% | 0,00% | - | - |
| | Bacilli | 0% | 0% | 0,00% | 0,00% | - | - |
| | Lactobacillales | 0% | 0% | 0,00% | 0,00% | - | - |
| | Clostridiales | 5% | 0% | 0,00% | 0,00% | 100% | - |
| | Thermoanaerobacteriales | 0% | 0% | 0,00% | 0,00% | - | - |
| | Rhodospirillales | 0% | 0% | 0,00% | 0,00% | - | - |
| | Rickettsiales | 5% | 0% | 10,00% | 0,00% | 100% | - |
| | Sphingomonadales | 0% | 0% | 0,00% | 0,00% | - | - |
| | Burkholderiales | 20% | 0% | 0,00% | 0,00% | 75% | - |

| | | | | | | | |
|---|---|---|---|---|---|---|---|
| | Nitrosomonadales | 0% | 0% | 0,00% | 0,00% | - | - |
| | Betaproteobacteria | 0% | 0% | 10,00% | 0,00% | - | - |
| | Desulfovibrionales | 0% | 0% | 5,00% | 0,00% | - | - |
| | Desulfuromonadales | 0% | 0% | 5,00% | 0,00% | - | - |
| | Campylobacterales | 5% | 0% | 0,00% | 0,00% | 100% | - |
| | Alteromonadales | 10% | 0% | 0,00% | 0,00% | 100% | - |
| | Enterobacteriales | 5% | 0% | 0,00% | 0,00% | 33% | - |
| | Pasteurellales | 20% | 0% | 0,00% | 0,00% | 67% | - |
| | Pseudomonadales | 15% | 0% | 0,00% | 0,00% | 100% | - |
| | Thiotrichales | 0% | 0% | 5,00% | 0,00% | - | - |
| | Vibrionales | 0% | 0% | 0,00% | 0,00% | - | - |
| | Xanthomonadales | 0% | 0% | 0,00% | 0,00% | - | - |
| | Gammaproteobacteria | 0% | 0% | 0,00% | 0,00% | - | - |
| | Spirochaetales | 35% | 0% | 0,00% | 0,00% | 100% | - |
| | Thermotogales | 20% | 0% | 0,00% | 0,00% | 100% | - |
| **Average** | | **7%** | **0%** | **2,43%** | **0%** | **74%** | **-** |

(-) Undefined value

| | | Sensitivity | | False Negative rate | | Specificity | |
|---|---|---|---|---|---|---|---|
| | | **TACOA** | **PhyloPhythia** | **TACOA** | **PhyloPythia** | **TACOA** | **yloPhythia** |
| Genus | Nitrosopumilus | 0% | 0% | 10,00% | 0,00% | - | - |
| | Metallosphaera | 0% | 0% | 0,00% | 0,00% | 0% | - |
| | Thermoproteus | 0% | 0% | 30,00% | 0,00% | - | - |
| | Halobacterium | 10% | 0% | 10,00% | 0,00% | 100% | - |
| | Mycobacterium | 0% | 0% | 0,00% | 0,00% | - | - |
| | Parabacteroides | 0% | 0% | 0,00% | 0,00% | - | - |
| | Porphyromonas | 0% | 0% | 0,00% | 0,00% | - | - |
| | Chlamydophila | 0% | 0% | 0,00% | 0,00% | 0% | - |
| | Chlamydia | 0% | 0% | 10,00% | 0,00% | - | - |
| | Chlorobium | 0% | 0% | 0,00% | 0,00% | - | - |
| | Chloroflexus | 0% | 0% | 10,00% | 0,00% | - | - |
| | Dehalococcoides | 30% | 0% | 0,00% | 0,00% | 100% | - |
| | Synechococcus | 0% | 0% | 0,00% | 0,00% | - | - |

| | | | | | | |
|---|---|---|---|---|---|---|
| Prochlorococcus | 0% | 0% | 0,00% | 0,00% | - | - |
| Bacillus | 0% | 0% | 0,00% | 0,00% | - | - |
| Lactobacillus | 0% | 0% | 0,00% | 0,00% | - | - |
| Streptococcus | 0% | 0% | 0,00% | 0,00% | - | - |
| Clostridium | 0% | 0% | 0,00% | 0,00% | - | - |
| Thermoanaerobacter | 0% | 0% | 0,00% | 0,00% | - | - |
| Magnetospirillum | 0% | 0% | 0,00% | 0,00% | 0% | - |
| Ehrlichia | 10% | 0% | 10,00% | 0,00% | 100% | - |
| Rickettsia | 0% | 0% | 0,00% | 0,00% | - | - |
| Erythrobacter | 0% | 0% | 0,00% | 0,00% | - | - |
| Sphingomonas | 0% | 0% | 0,00% | 0,00% | - | - |
| Sphingopyxis | 0% | 0% | 0,00% | 0,00% | - | - |
| Ralstonia | 0% | 0% | 0,00% | 0,00% | - | - |
| Nitrosomonas | 0% | 0% | 0,00% | 0,00% | - | - |
| Nitrosospira | 0% | 0% | 0,00% | 0,00% | - | - |
| Burkholderia | 50% | 0% | 0,00% | 0,00% | 100% | - |
| Desulfovibrio | 0% | 0% | 0,00% | 0,00% | - | - |
| Lawsonia | 0% | 0% | 0,00% | 0,00% | - | - |
| Geobacter | 0% | 0% | 0,00% | 0,00% | - | - |
| Campylobacter | 0% | 0% | 0,00% | 0,00% | - | - |
| Helicobacter | 0% | 0% | 0,00% | 0,00% | - | - |
| Shewanella | 0% | 0% | 0,00% | 0,00% | - | - |
| Serratia | 0% | 0% | 0,00% | 0,00% | - | - |

(-) Undefined value

| | | Sensitivity | | False Negative rate | | Specificity | |
|---|---|---|---|---|---|---|---|
| | | **TACOA** | **PhyloPhythia** | **TACOA** | **PhyloPythia** | **TACOA** | **yloPhythia** |
| Genus (Cont.) | Shigella | 0% | 0% | 0,00% | 0,00% | - | - |
| | Acinetobacter | 0% | 0% | 0,00% | 0,00% | - | - |
| | Psychrobacter | 0% | 0% | 0,00% | 0,00% | - | - |
| | Francisella | 0% | 0% | 0,00% | 0,00% | - | - |
| | Thiomicrospira | 0% | 0% | 0,00% | 0,00% | - | - |
| | Vibrio | 0% | 0% | 0,00% | 0,00% | - | - |
| | Xanthomonas | 0% | 0% | 0,00% | 0,00% | - | - |
| | Xylella | 0% | 0% | 0,00% | 0,00% | - | - |
| | Actinobacillus | 0% | 0% | 0,00% | 0,00% | - | - |
| | Azoarcus | 0% | 0% | 0,00% | 0,00% | - | - |
| | Leptospira | 30% | 0% | 0,00% | 0,00% | 100% | - |
| | Thermotoga | 10% | 0% | 0,00% | 0,00% | 100% | - |
| **Average** | | **3%** | **0%** | **1,67%** | **0%** | **67%** | **-** |

(-) Undefined value
