# Supplementary material for: TACOA – Taxonomic classification of environmental genomic fragments using a kernelized nearest neighbor approach
Source: BMC Bioinformatics. 2009 Feb 11;10:56. doi: 10.1186/1471-2105-10-56 (PMC2653487; doi:10.1186/1471-2105-10-56)
Supplement: Additional file 9 — Detailed accuracy obtained for genomic fragments of length 10 Kbp using TACOA and PhyloPythia classifiers. At each taxonomic rank, the classification accuracy (specificity and sensitivity) achieved for two different intrinsic classifiers: TACOA and PhyloPythia is given. The symbol (-) refers to the cases where the respective value cannot be mathematically defined. [file 1471-2105-10-56-S9.pdf]

Additional file 9. Detailed accuracy obtained for reads 10Kbp long using TACOA and PhyloPythia

		Sensitivity		False Negative rate		Specificity	
		**TACOA**	**PhyloPhythia**	**TACOA**	**PhyloPythia**	**TACOA**	**PhyloPhythia**
Superkingdom	Archaea	75%	70%	0,00%	27,50%	94%	76%
	Bacteria	70%	93%	0,34%	1,53%	100%	98%
**Average**		**73%**	**82%**	**0,17%**	**14,51%**	**97%**	**87%**
Phylum	Crenarchaeota	60%	40%	3,33%	30,00%	100%	100%
	Euryarchaeota	80%	60%	0,00%	40,00%	80%	27%
	Actinobacteria	25%	100%	0,00%	0,00%	100%	83%
	Bacteroidetes	0%	100%	0,00%	0,00%	-	100%
	Chlamydiae	90%	100%	0,00%	0,00%	100%	100%
	Chlorobi	0%	0%	40,00%	50,00%	-	-
	Chloroflexi	35%	0%	20,00%	5,00%	100%	100%
	Cyanobacteria	43%	93%	0,00%	5,00%	100%	97%
	Firmicutes	40%	100%	0,00%	0,00%	97%	92%
	Proteobacteria	59%	90%	0,63%	1,82%	94%	96%
	Spirochaetes	30%	50%	0,00%	0,00%	100%	100%
	Thermotogae	35%	0%	0,00%	45,00%	100%	-
**Average**		**41%**	**61%**	**5,33%**	**15%**	**97%**	**90%**
Class	Thermoprotei	53%	40%	0,00%	16,67%	100%	100%
	Halobacteria	80%	0%	0,00%	40,00%	100%	-
	Actinobacteria	25%	100%	0,00%	0,00%	100%	83%
	Bacteroidetes	0%	100%	0,00%	0,00%	-	100%
	Chlamydiae	100%	100%	0,00%	0,00%	56%	50%
	Chlorobia	0%	0%	35,00%	45,00%	-	-
	Chloroflexi	0%	0%	0,00%	0,00%	-	100%
	Dehalococcoidetes	70%	0%	0,00%	0,00%	100%	-
	Bacillales	0%	0%	55,00%	100,00%	-	-
	Bacilli	50%	95%	5,00%	0,00%	45%	48%
	Clostridia	15%	98%	0,00%	0,00%	100%	91%
	Alphaproteobacteria	40%	80%	0,00%	0,00%	100%	89%
	Betaproteobacteria	20%	65%	2,00%	0,00%	91%	53%

	Burkholderiales	0%	0%	0,00%	100,00%		-
	Deltaproteobacteria	13%	73%	2,50%	17,50%	100%	78%
	Epsilonproteobacteria	30%	95%	10,00%	0,00%	100%	83%
	Gammaproteobacteria	61%	88%	0,83%	6,15%	84%	87%
	Pasteurellales	0%	0%	40,00%	100,00%	-	-
	Rhodocyclales	0%	0%	5,00%	85,00%	-	-
	Spirochaetes	30%	45%	0,00%	5,00%	100%	100%
	Thermotogae	35%	0%	0,00%	45,00%	100%	-
**Average**		**30%**	**47%**	**7,40%**	**27%**	**91%**	**82%**

(-) Undefined value

		Sensitivity		False Negative rate		Specificity	
		**TACOA**	**PhyloPhythia**	**TACOA**	**PhyloPythia**	**TACOA**	**PhyloPhythia**
Order	Nitrosopumilales	0%	0%	10,00%	0,00%	-	-
	Sulfolobales	0%	0%	0,00%	0,00%	-	-
	Thermoproteales	90%	0%	0,00%	0,00%	100%	-
	Halobacteriales	10%	0%	0,00%	0,00%	100%	-
	Actinomycetales	0%	0%	0,00%	0,00%	-	-
	Bacteroidales	0%	0%	0,00%	0,00%	-	-
	Chlamydiales	100%	0%	0,00%	0,00%	56%	-
	Chlorobiales	0%	0%	10,00%	0,00%	-	-
	Chloroflexales	0%	0%	0,00%	0,00%	-	-
	Dehalococcoidetes	0%	0%	0,00%	0,00%	-	-
	Chroococcales	0%	0%	0,00%	0,00%	-	-
	Prochlorales	15%	0%	0,00%	0,00%	100%	-
	Bacilli	0%	0%	0,00%	0,00%	-	-
	Lactobacillales	50%	0%	0,00%	0,00%	100%	-
	Clostridiales	5%	0%	0,00%	0,00%	100%	-
	Thermoanaerobacteriales	0%	0%	0,00%	0,00%	-	-
	Rhodospirillales	0%	0%	0,00%	0,00%	-	-
	Rickettsiales	55%	0%	0,00%	0,00%	100%	-
	Sphingomonadales	0%	0%	6,67%	0,00%	-	-
	Burkholderiales	27%	0%	0,00%	0,00%	100%	-

	Nitrosomonadales	0%	0%	5,00%	0,00%	-	-
	Betaproteobacteria	0%	0%	0,00%	0,00%	-	-
	Desulfovibrionales	5%	0%	0,00%	0,00%	100%	-
	Desulfuromonadales	0%	0%	0,00%	0,00%	-	-
	Campylobacterales	15%	0%	0,00%	0,00%	100%	-
	Alteromonadales	45%	0%	0,00%	0,00%	100%	-
	Enterobacteriales	30%	0%	0,00%	0,00%	86%	-
	Pasteurellales	60%	0%	0,00%	0,00%	75%	-
	Pseudomonadales	10%	0%	0,00%	0,00%	100%	-
	Thiotrichales	10%	0%	0,00%	0,00%	100%	-
	Vibrionales	20%	0%	0,00%	0,00%	100%	-
	Xanthomonadales	5%	0%	0,00%	0,00%	100%	-
	Gammaproteobacteria	0%	0%	0,00%	0,00%	-	-
	Spirochaetales	30%	0%	0,00%	5,00%	100%	-
	Thermotogales	15%	0%	0,00%	0,00%	100%	-
**Average**		**17%**	**0%**	**0,90%**	**0%**	**96%**	**-**

(-) Undefined value

		Sensitivity		False Negative rate		Specificity	
		**TACOA**	**PhyloPhythia**	**TACOA**	**PhyloPythia**	**TACOA**	**PhyloPhythia**
Genus	Nitrosopumilus	0%	0%	10,00%	0,00%	-	-
	Metallosphaera	0%	0%	0,00%	0,00%	0%	-
	Thermoproteus	0%	0%	90,00%	0,00%	-	-
	Halobacterium	0%	0%	0,00%	0,00%	-	-
	Mycobacterium	0%	0%	0,00%	0,00%	-	-
	Parabacteroides	0%	0%	0,00%	0,00%	-	-
	Porphyromonas	0%	0%	0,00%	0,00%	-	-
	Chlamydophila	0%	0%	0,00%	0,00%	-	-
	Chlamydia	0%	0%	0,00%	0,00%	-	-
	Chlorobium	0%	0%	10,00%	0,00%	-	-
	Chloroflexus	0%	0%	0,00%	0,00%	-	-
	Dehalococcoides	70%	0%	0,00%	0,00%	100%	-
	Synechococcus	0%	0%	0,00%	0,00%	-	-

Prochlorococcus	0%	0%	0,00%	0,00%	-	-
Bacillus	0%	0%	0,00%	0,00%	-	-
Lactobacillus	0%	0%	0,00%	0,00%	-	-
Streptococcus	100%	0%	0,00%	0,00%	100%	-
Clostridium	5%	0%	0,00%	0,00%	100%	-
Thermoanaerobacter	0%	0%	0,00%	0,00%	-	-
Magnetospirillum	0%	0%	0,00%	0,00%	-	-
Ehrlichia	10%	0%	0,00%	0,00%	100%	-
Rickettsia	100%	0%	0,00%	0,00%	100%	-
Erythrobacter	0%	0%	0,00%	0,00%	-	-
Sphingomonas	0%	0%	0,00%	0,00%	-	-
Sphingopyxis	0%	0%	0,00%	0,00%	-	-
Ralstonia	0%	0%	0,00%	0,00%	-	-
Nitrosomonas	0%	0%	0,00%	0,00%	-	-
Nitrosospira	0%	0%	0,00%	0,00%	-	-
Burkholderia	80%	0%	0,00%	0,00%	100%	-
Desulfovibrio	10%	0%	0,00%	0,00%	100%	-
Lawsonia	0%	0%	0,00%	0,00%	-	-
Geobacter	0%	0%	0,00%	0,00%	-	-
Campylobacter	0%	0%	0,00%	0,00%	-	-
Helicobacter	10%	0%	0,00%	0,00%	100%	-
Shewanella	15%	0%	0,00%	0,00%	100%	-
Serratia	0%	0%	0,00%	0,00%	-	-

(-) Undefined value

		Sensitivity		False Negative rate		Specificity	
		TACOA	PhyloPhythia	TACOA	PhyloPythia	TACOA	PhyloPhythia
Genus (Cont.)	Shigella	0%	0%	0,00%	0,00%	-	-
	Acinetobacter	0%	0%	0,00%	0,00%	-	-
	Psychrobacter	20%	0%	0,00%	0,00%	100%	-
	Francisella	10%	0%	0,00%	0,00%	100%	-
	Thiomicrospira	0%	0%	0,00%	0,00%	-	-
	Vibrio	20%	0%	0,00%	0,00%	100%	-
	Xanthomonas	0%	0%	0,00%	0,00%	-	-
	Xylella	10%	0%	0,00%	0,00%	100%	-
	Actinobacillus	0%	0%	0,00%	0,00%	-	-
	Azoarcus	0%	0%	0,00%	0,00%	-	-
	Leptospira	30%	0%	0,00%	5,00%	100%	-
	Thermotoga	10%	0%	0,00%	0,00%	100%	-
**Average**		**10%**	**0%**	**2,29%**	**0,10%**	**94%**	**-**

(-) Undefined value
